# Zinc Oxide Nanoparticles Affect Early Seedlings’ Growth and Polar Metabolite Profiles of Pea (*Pisum sativum* L.) and Wheat (*Triticum aestivum* L.)

**DOI:** 10.3390/ijms241914992

**Published:** 2023-10-08

**Authors:** Karolina Stałanowska, Joanna Szablińska-Piernik, Adam Okorski, Lesław B. Lahuta

**Affiliations:** 1 Department of Plant Physiology, Genetics and Biotechnology, University of Warmia and Mazury in Olsztyn, Oczapowskiego 1A, 10-719 Olsztyn, Poland; karolina.stalanowska@uwm.edu.pl (K.S.); joanna.szablinska@uwm.edu.pl (J.S.-P.); 2 Department of Entomology, Phytopathology and Molecular Diagnostics, University of Warmia and Mazury in Olsztyn, Pl. Łódzki 5, 10-727 Olsztyn, Poland

**Keywords:** pea, wheat, seedling, zinc oxide nanoparticles, polar metabolite profiles

## Abstract

The growing interest in the use of zinc oxide nanoparticles (ZnO NPs) in agriculture creates a risk of soil contamination with ZnO NPs, which can lead to phytotoxic effects on germinating seeds and seedlings. In the present study, the susceptibility of germinating seeds/seedlings of pea and wheat to ZnO NPs of various sizes (≤50 and ≤100 nm) applied at concentrations in the range of 100–1000 mg/L was compared. Changes in metabolic profiles in seedlings were analyzed by GC and GC-MS methods. The size-dependent harmful effect of ZnO NPs on the seedling’s growth was revealed. The more toxic ZnO NPs (50 nm) at the lowest concentration (100 mg/L) caused a 2-fold decrease in the length of the wheat roots. In peas, the root elongation was slowed down by 20–30% only at 1000 mg/L ZnO NPs. The metabolic response to ZnO NPs, common for all tested cultivars of pea and wheat, was a significant increase in sucrose (in roots and shoots) and GABA (in roots). In pea seedlings, an increased content of metabolites involved in the aspartate–glutamate pathway and the TCA cycle (citrate, malate) was found, while in wheat, the content of total amino acids (in all tissues) and malate (in roots) decreased. Moreover, a decrease in products of starch hydrolysis (maltose and glucose) in wheat endosperm indicates the disturbances in starch mobilization.

## 1. Introduction

Zinc oxide nanoparticles (ZnO NPs) have a lot of applications in many industry sectors, e.g., in electronics and electrical engineering, in medicine (due to their anti-bacterial, anti-tumor, and anti-inflammation properties), and can also serve as UV blockers in clothes or sunscreens [1,2,3]. Moreover, due to their biocidal properties, ZnO NPs have great potential for agricultural applications, especially as a novel compound of pesticides. ZnO NPs decrease *Fusarium proliferatum* growth in maize (*Zea mays* L.) grains [4], restrain *Fusarium oxysporum* growth, and suppress Fusarium wilt disease in eggplant (*Solanum melongena* L.) [5], and they can also be used as a foliar spray to suppress infections of *Erysiphe betae*, a causal agent of powdery mildew of sugar beet (*Beta vulgaris* L.) [6]. It has also been reported that ZnO NPs significantly inhibit the growth of some plant pathogenic bacteria, such as *Xanthomonas oryzae* pv. *oryzae*, *Xanthomonas axonopodis* pv. *citri*, and *Ralstonia solanacearum* [7]. ZnO NPs can also protect plants against various abiotic stresses, e.g., heavy metal, drought, or salinity stress, alleviating their negative effects [8]. Exposure of cotton (*Gossypium hirsutum* L.) to ZnO NPs alleviated the phytotoxic effect of cadmium (Cd) and lead (Pb), whereas foliar application of ZnO NPs alleviated Cd stress in tobacco (*Nicotiana tabacum* L.) [9]. In maize, ZnO NPs promoted drought stress tolerance and increased the activity of enzymes involved in primary carbohydrate metabolism (UDP-glucose pyrophosphorylase, phosphoglucose isomerase, and cytoplasmic invertase) [10]. Fazian et al. [11] showed that the foliar application of ZnO NPs to tomato (*Lycopersicon esculentum* Mill.) can promote salt-stress tolerance. An increase in antioxidative enzyme activities, such as peroxidase (POX), superoxide dismutase (SOD), and catalase (CAT), was also observed. It has been suggested that ZnO NPs improve plant tolerance to various stresses by increasing the activity of antioxidant enzymes and regulating primary carbohydrate metabolism, which has a positive impact on nutrient uptake and plant growth [8]. ZnO NPs also have a positive effect on seed germination, seedling growth, and an increase in plant biomass and yield [12,13,14]. Thus, it is considered a potential nano-fertilizer, which can also counteract zinc deficiency in the soil [15]. Nano-fertilizers are a step forward to precision and less expensive agriculture. They are more effective than standard fertilizers (less amount is needed to achieve the same results), and the release of nutrients is slower and more controlled [1,8,16].

However, adverse effects of ZnO NPs are also observed, depending on their concentration and physicochemical properties like size, shape, surface charge, surface coating, dissolution, and agglomeration [17,18,19]. Moreover, their phytotoxicity is plant species-specific and connected with nanoparticle uptake and transfer capacity in plants [7,19]. ZnO NPs can inhibit seedling growth, reduce plant biomass, decrease chlorophyll content, and cause oxidative damage, DNA damage, chromosome aberration, disruption of the cell membrane, and disturbance in the cell cycle [20,21,22]. There is a fine line between the toxic and beneficial effects of ZnO NPs due to the easy metabolization of Zn ions by plants [22].

ZnO NPs can be simply absorbed by plants, which leads to their overaccumulation and phytotoxic effect, presumably due to the release of excess Zn^2+^ ions. However, the toxicity of nanoparticles themselves was also reported [20,23]. ZnO NPs and zinc ions released from nanoparticles can be transported axially through the phloem and xylem but also radially—via symplastic or apoplastic pathway [24,25]. Da Cruz et al. [24] revealed that in common bean (*Phaseolus vulgaris* L.), elevated expression of genes encoding metal tonoplast-localized carriers was observed after ZnO NPs and ZnSO_4_ exposure. This suggests that an excess of ZnO NPs and/or Zn^2+^ released from nanoparticles can be stored in vacuoles to maintain the Zn balance.

Optimal concentrations of Zn are crucial for the proper functioning of plant cells. It is a prosthetic group of many enzymes, such as dehydrogenases, aldolases, isomerases, transphosphorylases, RNA, and DNA polymerases. In addition, zinc ions stabilize the zinc finger domain, which is observed in many proteins (such as transcription factors) and is responsible for proper protein–nucleic acid and protein–protein interactions [26,27]. Thus, zinc is essential for gene expression and regulation. It has also been reported that zinc is required for auxin production, as it is needed for the synthesis of tryptophan, which is a precursor of auxin (indolyl-acetic acid, IAA). Zinc is also involved in signal transduction, maintaining the integrity of the cellular membranes, carbohydrate and lipid metabolism, photosynthetic metabolism, and plant defense responses [26,27,28]. Therefore, zinc deficiency causes inhibition of RNA and protein synthesis, as well as chlorosis, growth retardation, wilting, and rolling of leaves and stems [15].

Due to the increasingly common use of ZnO NPs, it is particularly important to test the sensitivity of important crop plants to nanoparticles, including wheat (*Triticum aestivum* L.) and pea (*Pisum sativum* L.), one of the most important cereals and legume species in the human diet and livestock feed [29,30]. The application of ZnO NPs (13 nm) at concentrations of 10–100 mg/L had a positive effect on wheat seed germination and seedling development [31]. A more toxic effect was observed for larger ZnO NPs (20 nm)—seedlings root and coleoptile length were inhibited after nanoparticle treatment at doses over 50 and 100 mg/L, respectively. However, ZnSO_4_ used at the corresponding concentration range was more toxic than nanoparticles [32]. Pea cultivation in soil enriched with ZnO NPs at concentrations 125, 250, and 500 mg/kg resulted in pea root increased elongation. Some alterations in the antioxidant system were also observed [29]. Huang et al. [33] showed that pea seeds exposure to ZnO NPs at concentrations 500–1000 mg/L did not influence seed germination but negatively affected root elongation. Nanoparticles also act on pea-rhizobia symbiosis by decreasing nodulation (ZnO NPs at concentrations 250 and 750 mg/L), which affects nitrogen fixation [33].

There are still limited published data regarding the changes in pea and wheat metabolome/metabolite profiles under ZnO NPs treatment [34], especially at the earliest stages of development (seed germination/seedling growth), which are crucial for further plant growth and yield. Considering the potential use of ZnO NPs in agriculture as nano-fertilizers or nano-pesticides, used as foliar sprays, or in seed nano-priming, such investigations are needed to better understand the phytotoxic properties of nanoparticles and their possible mechanisms of influence on seed germination, seedlings growth, and their primary metabolism. Therefore, in the present study, the effects of commercially available, chemically synthesized ZnO NPs on (a) germination and early seedlings development of garden pea (*Pisum sativum* L.) and wheat (*Triticum aestivum* L.), two important crops of different clades (dicots and monocots, respectively), and (b) changes in the polar metabolite profiles of seedlings were compared. To the best of our knowledge, this is the first study to analyze the changes in the polar metabolite profiles of pea and wheat seedlings induced by continuous ZnO NPs treatment. These changes may underlie the different sensitivities of wheat and pea to ZnO NPs.

## 2. Results

### 2.1. The Preliminary Study

In the first part of the preliminary study, the phytotoxic concentrations of ZnO NPs to seed germination and early seedling development of peas and wheat were investigated. The 3 and 4 days of incubation (for wheat and peas, respectively) in the suspension of ZnO NPs (≤50 nm) at concentrations 20, 50, 250, 500, and 1000 mg/L did not affect the germination of pea and wheat seeds (of both cultivars). No statistical differences (*p* < 0.05) were observed in the length, FW, and DW of seedlings of cv. Nemo (Supplementary Materials, Table S1), whereas they were found in cv. Tarchalska, at elevated concentrations of ZnO NPs (≥250 mg/L, Table S1). In wheat (both cultivars), the root length and FW decreased with increasing concentrations of ZnO NPs, whereas no differences were found in coleoptiles (Table S2).

Subsequently, the effects of ZnO NPs of different sizes (ø < 50 nm and <100 nm) were compared in pea cv. Tarchalska and wheat cv. Ostka Strzelecka. Nanoparticles (of both sizes) at concentrations of 100, 250, and 1000 mg/L did not affect the germination of pea and wheat seeds (Tables S3 and S4). Moreover, regardless of their size, ZnO NPs inhibited the growth of the pea seedlings. However, smaller nanoparticles caused stronger inhibition of epicotyl elongation, compared with root. The opposite effect was observed in seedlings grown in the suspension of larger ZnO NPs (Table S3). In wheat, smaller ZnO NPs caused stronger inhibition of root growth—the length of the primary root was decreased by 52, 66, and 71% (at concentrations of 100, 250, and 1000 mg/L, respectively), whereas under treatment with larger ZnO NPs (<100 nm) the length of the root was lowered by 50, 59, and 68% (Table S4).

The above results led to the selection of smaller ZnO NPs (<50 nm) at concentrations of 100, 250, and 1000 mg/L for the main experiment focusing on the comparisons of changes in metabolome in pea and wheat seedlings in response to ZnO NPs.

### 2.2. The Effect of ZnO NPs on Seed Germinability and Seedling Growth

**Figure 1:**
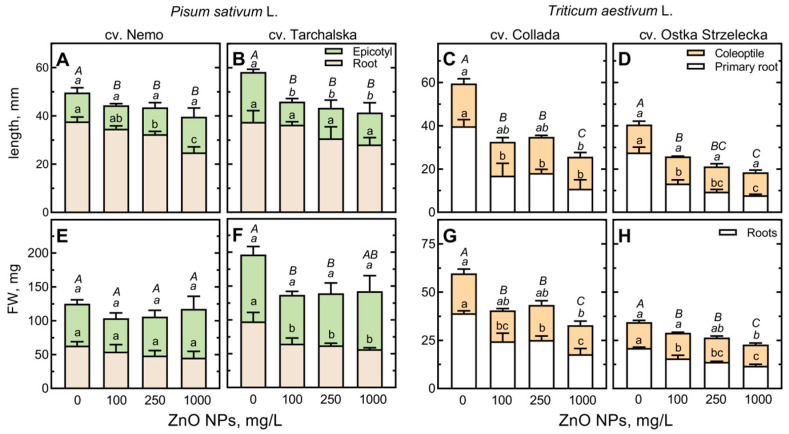
The effects of ZnO NPs (at concentrations of 0, 100, 250, and 1000 mg/L) on the length and fresh weight (FW) of roots and shoots (epicotyl of pea and coleoptile of wheat) of 4-day-old seedlings of pea (*Pisum sativum* L.) cv. Nemo (**A**,**E**) and cv. Tarchalska (**B**,**F**) and 3-day-old seedlings of wheat (*Triticum aestivum* L.) cv. Collada (**C**,**G**) and cv. Ostka Strzelecka (**D**,**H**). Values are means of 3 replicates + SD. The same lowercase letters (for shoots and roots, separately) and uppercase letters (for total seedlings length) above the bars indicate no significant (*p* < 0.05) differences according to the ANOVA test and post hoc Tukey’s corrections.

ZnO nanoparticles at concentrations in the range of 100–1000 mg/L did not negatively affect the germination of pea and wheat seeds. Wheat grains (both cultivars) finished germination at 95–100% after the first day, both in water and suspensions of ZnO NPs. The rate of pea seed germination was slightly slowed—after 24 h of hydration in water, 60% of the seeds completed germination (the radicle protruded the seed coat and reached a length of 2–3 mm), while after another 24 h, the germinability increased to 100%. Zinc oxide nanoparticles at a concentration of 100 mg/L accelerated the completion of seed germination in both pea cultivars (from 60 to 80%), while higher concentrations had no significant effect on this process. However, the growth and development of seedlings of both species were significantly (*p* < 0.05) affected by ZnO NPs (along with increasing concentrations). In peas, ZnO NPs slightly decreased the elongation of root (cv. Nemo) or epicotyl (cv. Tarchalska, Figure 1A,B), whereas in wheat, nanoparticles dramatically inhibited the elongation and fresh weight gain in both roots and coleoptiles (Figure 1C,D,G,H and Figure S1).

The morphological deformations of seedlings were not observed. Noticeably, in Petri dishes with wheat seeds germinating in ZnO NP suspensions, white precipitates appeared along with increasing concentrations of ZnO NPs (Figure S1). Such precipitates were not noted in dishes with germinating pea seeds (data not presented).

### 2.3. The Composition and Content of Polar Metabolites in Control Seedlings of Pea and Wheat

In seedlings and cotyledons of peas, 39 and 34 polar metabolites were identified, respectively (Tables S5–S7), whereas in seedlings and endosperms of wheat, 34 and 35 were identified, respectively (Tables S8–S10). In both analyzed species, the same set of polar metabolites was identified in the tissues of growing roots and shoots, i.e., 5 soluble carbohydrates (fructose, glucose, galactose, *myo*-inositol, and sucrose), 17 amino acids (alanine, asparagine, aspartic acid, γ-aminobutyric acid, glutamic acid, glutamine, hydroxyproline, isoleucine, leucine, lysine, phenylalanine, proline, serine, threonine, tyrosine, and valine), 6 organic acids (citric, fumaric, lactic, malic, and propionic acids), phosphoric acid, and urea. In seedlings of peas, non-proteinogenic amino acids (homoserine and b-alanine) and an additional six less abundant organic acids (acetic, butyric, glutaric, malonic, oxalic, and succinic acid) were detected. Pea cotyledons also contained raffinose and stachyose (oligosaccharides absent in wheat seedlings). The polar metabolites present in seedlings of wheat, but not detected in peas, were 1-kestose (tri-saccharide), methionine, maltose, and maltotriose (in endosperm only).

**Figure 2:**
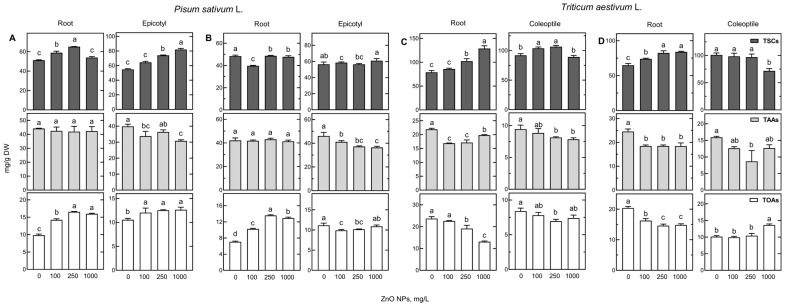
The concentration of total soluble carbohydrates (TSCs), total amino acids (TAAs), and total organic acids (TOAs) in the roots and epicotyls of 4-day-old seedlings of pea (*Pisum sativum* L.) cv. Nemo (**A**) and cv. Tarchalska (**B**) and roots and coleoptiles of 3-day-old seedlings of wheat (*Triticum aestivum* L.) cv. Collada (**C**) and cv. Ostka Strzelecka (**D**). Values (in mg/g DW) are means of 3 replicates + SD. The same letters above the bars indicate no significant (*p* < 0.05) differences according to the ANOVA test and post hoc Tukey’s corrections.

The concentration of total identified polar metabolites (TIPMs) was similar in the corresponding pea and wheat organs, remaining 2-fold lower in storage tissues (cotyledons and endosperm) than in the growing parts of seedlings, regardless of seedling treatment (Figure 2, Tables S5–S10). The most prevalent fraction in the polar metabolites of both species was soluble carbohydrates (Figure 2).

The concentration of total soluble carbohydrates (TSCs) in epicotyls and roots of control seedlings of pea was slightly higher than that in cotyledons (48–56 and 41–46 mg/g DW, respectively) and accounted for 44 to 48% of TIPMs, while the participation of sugars in TIPMs in cotyledons was much higher (71–77%). In epicotyl and root, the second major fraction among polar metabolites was amino acids (total 42–46 mg/g DW), accounting for 35–40% of TIPMs (Figure 2A,B; Tables S5 and S6). In cotyledons, the concentrations of total amino acids (TAAs) and total organic acids (TOAs) were much lower (8–11 and 3–4.5 mg/g DW, respectively) than in growing tissues (Table S7). Moreover, in cotyledons, the concentration of phosphoric acid was much lower than in seedlings (ca. 2 and 10 mg/g DW, respectively, Tables S5–S7).

In control wheat seedlings, soluble carbohydrates (65–109 mg/g DW) constituted over half of the polar metabolites in roots, sharing 55–60% of TIPMs, and 76–80% of TIPMs in coleoptiles (92–109 mg/g DW, Figure 2C,D; Tables S8 and S9). In the endosperm, the participation of TSCs in TIPMs was much higher, up to 91–93% (Table S10). The concentration of TAAs in roots was similar in both wheat cultivars (22–25 mg/g DW) and higher than that in coleoptiles (9.5 and 16 mg/g DW). The concentration of TOAs in roots was as high (22–24 mg/g DW) as TAAs and more than twice as high as in the coleoptile (Figure 2). In endosperms, the levels of TAAs and TOAs were much lower (2.8–2.9 and 0.91–0.95 mg/g DW, respectively) than in growing tissues (Table S10).

Among the polar metabolites detected in the growing tissues of control seedlings of pea and wheat (developed in the water), sucrose, glucose, galactose, fructose, asparagine, citrate, and malate were the predominant metabolites. A high content of homoserines was detected in peas, whereas the roots of wheat seedlings contained much more glutamine and hydroxyproline than pea seedlings. Additionally, 1-kestose (a short-chain fructan) was found in wheat tissues (Table 1). Tissues also contained a considerable amount of phosphoric acid (5–10 mg/g DW). Moreover, in wheat coleoptile, elevated levels of glucose and fructose coincided with a lowered level of sucrose. Such a negative relationship was not found in peas.

In cotyledons of a pea, the major metabolite was sucrose (37–42 mg/g DW, Table S7), whereas in wheat endosperm, maltose (34 and 26 mg/g DW in cv. Collada and Ostka Strzelecka, respectively) and glucose (9.3 and 6.5 mg/g DW) dominated (Table S10). Small amounts of raffinose family oligosaccharides (raffinose and stachyose) were present (<2 mg/g DW, Table S3) only in pea cotyledons.

In seedlings developing in ZnO NP suspensions, the concentrations of many metabolites changed (Tables S5–S10), leading to differences in the metabolic profiles and the concentrations of polar metabolite fractions (Figure 2). However, the shift in changes was not uniform, except for a similar trend in the increase of TIPMs in the roots of both wheat cultivars (Figure 2). This was a result of the elevated levels of TSCs, along with the increasing concentration of ZnO NPs.

### 2.4. The PCA of Metabolic Profiles of Seedlings under ZnO NPs Treatment

**Figure 3:**
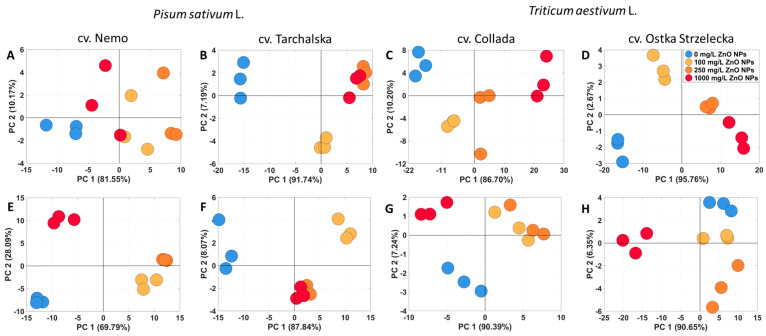
PCA of metabolic profiles of roots (**A**,**B**) and epicotyls (**E**,**F**) of 4-day-old seedlings of pea (*Pisum sativum* L.) and roots (**C**,**D**) and coleoptile (**G**,**H**) of 3-day-old seedlings of wheat (*Triticum aestivum* L.) developed in suspension of ZnO NPs at 0, 100, 250, and 1000 mg/L.

The principal component analysis (PCA) of polar metabolites in root/epicotyl of pea and roots/coleoptile of wheat seedlings showed a clear separation of control samples from those treated with ZnO NPs (Figure 3). The shift in the distribution of the samples changed with the increasing concentration of ZnO NPs. This was especially noted for the roots of pea cv. Tarchalska and roots of both wheat cultivars, where PC1 shared 91.74, 86.70, and 95.76% of variability, respectively (Figure 3B–D). In wheat, the control samples of roots and those treated with 100 mg/L ZnO NPs were not only separated from those treated with ZnO NPs at higher concentrations but also from each other according to PC2 (10.20 and 2.67% of variability for cv. Collada and Ostka Strzelecka, respectively) (Figure 3C,D). Pea epicotyl cv. Nemo samples and wheat coleoptile samples of both cultivars, after treatment with ZnO NPs at concentrations 100 and 250 mg/L, were grouped together, apart from the control and samples treated with higher nanoparticle concentrations (Figure 3E,G,H). In contrast, pea epicotyl cv. Tarchalska samples treated with 250 and 1000 mg/L ZnO NPs were grouped together (Figure 3F).

The distribution of seedling samples of both species was influenced mainly by changes in the concentrations of sucrose and glucose, as revealed by the PCA loading plots (Figure S2). Additionally, samples of pea seedlings were differentiated by galactose, homoserine, citric acid, and malic acid, while those of wheat by fructose and 1-kestose (Figure S2). In cotyledons of peas, sucrose and β-alanine were the major metabolites affecting the separation of samples (Figure S3). However, the pattern of sample distribution was not so clear (Figure S3), compared with those for seedlings (Figure 3). In wheat, endosperms of control seedlings were focused on the right from PC1 (sharing 96–98% of variability), whereas those treated with ZnO NPs were mostly on the left (Figure S4). This was a result of changes in the concentrations of maltose and sucrose (Figure S4).

### 2.5. Changes of Polar Metabolites in Seedlings’ Response to ZnO NPs

#### 2.5.1. Metabolites Mostly Differentiate Samples According to the Results of PCA

**Figure 4:**
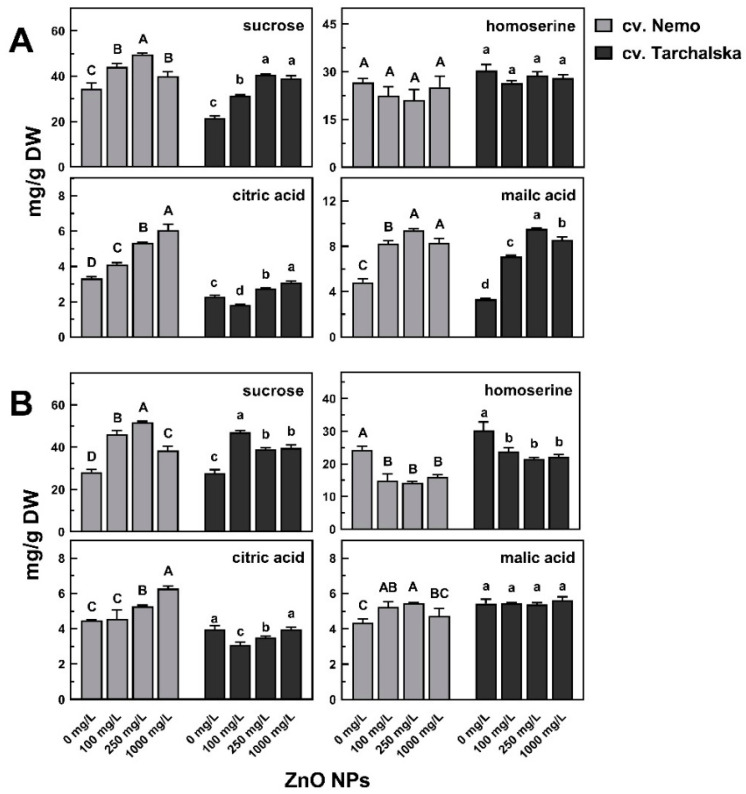
The effect of ZnO NPs on the concentration of metabolites mostly differentiating root (**A**) and epicotyl (**B**) samples (according to PCA analysis—Figure 3 and Figure S2) of 4-day-old seedlings of pea (*Pisum sativum* L.) cv. Nemo and cv. Tarchalska. Means of 3 replicates + SD. The same letters (A–D and a–d separately for cv. Nemo and Tarchalska, respectively) by the values indicate no significant (*p* < 0.05) differences based on ANOVA analysis and Tukey’s post hoc corrections.

The concentration of sucrose, the carbohydrate most differentiating pea samples (Figure S2A,B,E,F), and the quantitatively major soluble carbohydrate in seedlings of both pea cultivars, was significantly (*p* < 0.05) higher in the roots and epicotyls of seedlings developing in ZnO NPs compared with the control (Tables S1 and S2). The highest concentration of sucrose was found in roots treated with ZnO NPs at 250 mg/L (49.70 and 40.63 mg/g DW in cv. Nemo and Tarchalska, respectively), while in epicotyls at 100 mg/L (46.94 mg/g DW in cv. Tarchalska) or 250 mg/L (52.74 mg/g DW in cv. Nemo, Figure 4).

Although the elevated level of sucrose was accompanied by a decrease in monosaccharides, i.e., fructose, galactose, and glucose (Tables S5 and S6), the negative correlations between changes in the concentrations of sucrose and monosaccharides (separately glucose, galactose and fructose) were found only in cv. Tarchalska in both roots (*r* = −0.88, *r* = −0.88 and *r* = −0.86, Table S11) and epicotyls (*r* = −0.90, *r* = −0.87 and *r* = −0.80, Table S11).

Among the amino acids, homoserine was dominant, regardless of pea seedlings treatment (Figure 4A, Tables S5 and S6). Its concentration was only slightly decreased in the roots of seedlings developing in ZnO NPs. However, it was significantly lowered by ZnO NPs in epicotyls, by more than 35 and 22% in cv. Nemo and cv. Tarchalska, respectively (Figure 4B, Tables S5 and S6).

The most abundant organic acids were citric and malic acids. Their concentrations significantly increased in the roots of both pea cultivars (Figure 4A) and in epicotyls of cv. Nemo (Figure 4B). The concentration of citric acid in roots and epicotyls of cv. Nemo increased along with the increasing concentrations of ZnO NPs (up to ca. 6 mg/g DW). The maximum levels of malic acid in roots and epicotyls (9.39 and 5.45 mg/g DW, respectively) were found in seedlings developed in ZnO NPs at 250 mg/L. In cv. Tarchalska, roots of seedlings treated with ZnO NPs (at 250 and 1000 mg/L) contained only slightly more citric acid than the control, but the concentration of malic acid was dramatically increased (2–3-fold, Figure 4A).

**Figure 5:**
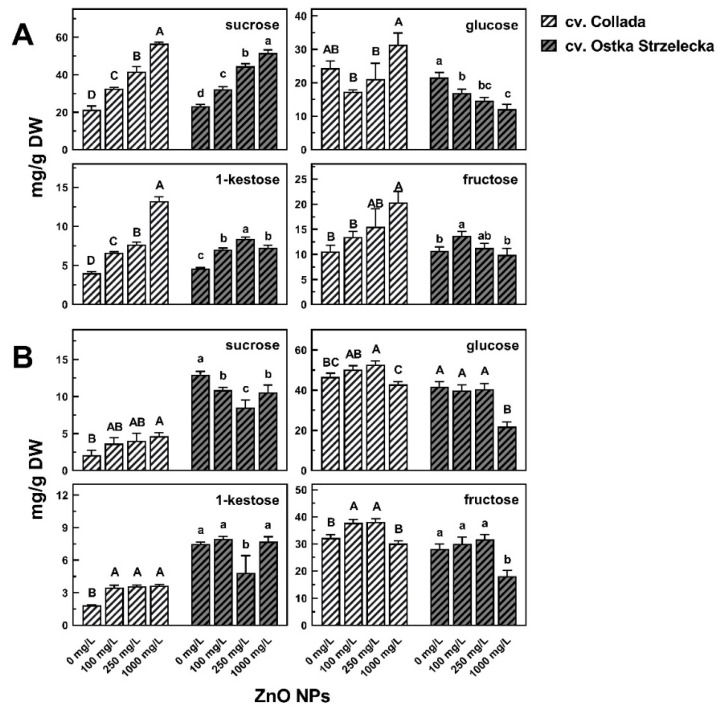
The effect of ZnO NPs on the concentration of metabolites mostly differentiating roots (**A**) and coleoptile (**B**) samples (according to PCA analysis—Figure 4 and Figure S1) of 3-day-old wheat seedlings cv. Collada and cv. Ostka Strzelecka. Means of 3 replicates + SD. The same letters (A–D and a–d separately for cv. Collada and Ostka Strzelecka, respectively) by the values indicate no significant differences (*p* < 0.05) based on ANOVA analysis and Tukey’s post hoc corrections.

In wheat roots, the most dominant soluble sugars were sucrose and glucose, whereas in coleoptile—glucose and fructose (Figure 5; Tables S8 and S9). Changes in their concentrations were the major reasons for shifts in the metabolic profiles of seedling samples under ZnO NPs treatment (Figure S2C,D,G,H). In the roots of both wheat cultivars, the amount of sucrose increased along with the increasing concentrations of ZnO NPs (from 21.36 to 56.61 mg/g DW in cv. Collada and from 23.31 to 51.61 mg/g DW in cv. Ostka Strzelecka) (Figure 5A).

The effect of ZnO NPs on the concentration of sucrose in coleoptiles was different, causing a 2-fold increase in cv. Collada, while a decrease in cv. Ostka Strzelecka (Figure 5B). Besides sucrose, root tissues (both cultivars) under ZnO NPs treatment accumulated 1-kestose. The concentration of this fructan was also duplicated in coleoptiles of cv. Collada (Figure 5). However, the correlations between changes in the concentrations of sucrose plus 1-kestose and monosaccharides (fructose plus glucose) in roots were opposite, i.e., positive in cv. Collada and negative in cv. Ostka Strzelecka (*r* = 0.72 and *r* = −0.90, respectively, Table S12). No correlations were found in coleoptiles (Table S12).

#### 2.5.2. Other Metabolites

The concentrations of some amino acids were significantly affected by ZnO NPs in seedlings of both studied species. In peas, the roots of seedlings developed in ZnO NPs contained more aspartic acid, β-alanine, γ-aminobutyric acid (GABA), and glutamic acid than the roots of control seedlings (Tables S5 and S6). Similar differences were found in epicotyls, but only in seedlings that developed in ZnO NPs at concentrations of 100 and 250 mg/L. Moreover, a decrease in asparagine was found (Tables S5 and S6). It should be noted that ZnO NPs (at applied concentrations) had no effect on the concentration of TSCs (among them, sucrose, raffinose, stachyose, and *myo*-inositol) in pea cotyledons (Table S3).

In wheat, the concentrations of asparagine and glutamine (the most dominant amino acids) in roots decreased with increasing concentrations of ZnO NPs. Asparagine decreased by ca. 45 and 50% and glutamine by 40 and 75% in cv. Collada and cv. Ostka Strzelecka, respectively. In coleoptiles, the concentrations of asparagine and valine also decreased, as in roots (asparagine by ca. 30% in cv. Collada and by 50–75% in cv. Ostka Strzelecka), whereas the concentration of valine decreased by 20% in coleoptiles of both cultivars (Tables S8 and S9). Additionally, the decrease in isoleucine and valine (by ca. 20–45% and by 30–40%, respectively) and accumulation of GABA were also observed in the roots of both wheat cultivars (Tables S8 and S9).

In wheat endosperms, ZnO NPs caused a decrease in most fractions of the identified metabolites (Table S10). The concentration of maltose (predominant soluble carbohydrate, sharing more than 50% of TSCs in endosperm) decreased with increasing ZnO NPs concentration. Concomitantly, a decrease in maltotriose and a slight increase in sucrose were observed (Table S10).

## 3. Discussion

### 3.1. Physiological Effects of ZnO NPs

The dual effects of zinc oxide nanoparticles on seed germination and plant growth depend on the nanoparticles’ physicochemical properties, concentration, and plant species [19]. Our findings suggest that ZnO NPs at concentrations up to 1000 mg/L have no negative impact on the germination of wheat and pea seeds. Consistent results with ZnO NPs have been documented for various plant species, i.e., pea [33], wheat [30], radish, rape, ryegrass, lettuce, corn, and cucumber [35]. 

The inhibitory effect of ZnO NPs on the growth of pea and wheat seedlings presented in this study (Figure 1 and Figure S1) was probably a result of the over-uptake of nanoparticles by the roots. In our study, the uptake of ZnO NPs was not analyzed, but the reactions of seedlings to increasing concentrations of ZnO NPs (at both physiological and metabolomic levels, Figure 1 and Figure S1, Tables S5–S10) seem to be an indirect confirmation of this process. Similar findings were presented by Huang et al. [33] in peas and by Srivastav et al. [30] in wheat. In their investigations, they used the same ZnO NPs manufactured by Sigma-Aldrich as in the present study. Huang et al. [33] showed that 24 h of pea seed imbibition in ZnO NPs at 250 mg/L resulted in inhibition of pea seedling root elongation during the next few days of seedling development in the absence of ZnO NPs. In our study, seeds germinated, and seedlings grew in the constant presence of ZnO NPs; thus, this long exposure was a reason for seedlings’ growth retardation at the lower concentration of ZnO NPs (100 mg/L). Similarly, the ZnO NPs at 100 mg/L dramatically decreased the growth of wheat seedlings (Figure 1). However, Srivastav et al. [30] found a remarkable decrease in growth and biomass accumulation in seedlings of maize and wheat even at a 2-fold higher concentration of ZnO NPs (200 mg/L). Moreover, in the above-mentioned studies [30,33], an accumulation of higher amounts of zinc in roots than in shoots was documented.

In our study, ZnO NPs showed harmful effects at all tested concentrations on both species, but it was the most noticeable at the concentration of 1000 mg/L (Figure 1). Moreover, the inhibitory impact on root elongation was evident in wheat seedlings—the radicle length of both wheat cultivars was decreased by over 50% at 100 mg/L (Figure 1C,D). In peas, the root length was decreased by 25 and 34% (in cv. Tarchalska and Nemo, respectively) even at a 10-fold higher concentration of ZnO NPs (Figure 1A,B). The high susceptibility of wheat roots (and to a lesser extent also coleoptile) to other metal nanoparticles was documented earlier [36,37,38,39]. In our previous study, dead cells and accumulation of reactive oxygen species (ROS) were observed in the wheat root meristem and in the elongation zone under treatment with silver nanoparticles, applied at several-fold lower concentrations. Moreover, the visible symptom of cell deterioration was the browning of the root tip [38,40]. In the present study, such an effect was not observed, presumably due to the lower toxicity of ZnO NPs than AgNPs.

The inhibition of root growth (Figure 1) was not obviously accompanied by a decrease in the elongation of coleoptile (found only in cv. Collada) and epicotyl (found in cv. Tarchalska). The absorbed ZnO NPs (and zinc ions Zn^2+^ released from nanoparticles) can be transported from roots to shoot not only through the xylem but also via symplastic or apoplastic pathways [24,25]. Thus, the different effects of ZnO NPs on the elongation of roots/shoots of peas and wheat could be a result of differences in the transport of ZnO NPs/Zn^2+^ ions in seedlings. Moreover, zinc or specific metal transporters might be involved in ZnO NPs transport in both species [24].

On the other hand, zinc is crucial for auxin synthesis, and its excess can reduce IAA accumulation and transport in a dose-dependent manner. In *Arabidopsis*, zinc at high concentrations (over 100 µM ZnSO_4_), besides the reduction of primary root length (caused by inhibition of meristematic cell division), also negatively affected the total number and the density of lateral roots [41]. Therefore, the effects of ZnO NPs on seedlings’ growth can be also a result of an imbalance in hormonal homeostasis [42].

### 3.2. Changes in Polar Metabolite Profile after ZnO NPs Treatment

In the first stages of seedling development, reserve materials stored in seeds (carbohydrates, proteins, and lipids) are mobilized and utilized by growing seedlings [43]. In pea cotyledons, the content of all fractions of identified polar metabolites did not change under ZnO NPs treatment, which suggests that mobilization and utilization of storage materials in cotyledons were not affected by nanoparticles (Table S7). However, some changes in wheat endosperm (especially, soluble sugar content) occurred under ZnO NPs treatment in a dose-dependent manner (Table S10). The decreased content of maltose and maltotriose in endosperm might be related to the inhibition of α-amylase activity [30] and, therefore, inhibition of starch degradation [44]. Such inhibitory properties of ZnO NPs were previously reported in germinating seeds/seedlings of wheat [30] and also in vitro study of fibroblast cell lines [45].

Shifts in the TIPMs profiles of wheat and pea seedlings caused by ZnO NPs were related to changes in the concentration of soluble carbohydrates, especially sucrose (Figure 2, Figure 4 and Figure 5, Tables S5–S10), accumulated under ZnO NPs treatment in roots and epicotyl/coleoptile (Figure 4 and Figure 5). Sucrose accumulation was also observed in the roots of wheat seedlings germinated and developed in Ag NPs solutions [38] and in tomato roots after foliar spray with ZnO NPs [46]. It should be noted that the sources of sucrose in germinating seeds of wheat and peas are not the same. In wheat, sucrose is synthesized in the scutellum (from glucose and maltose, a final product of starch hydrolysis in endosperm) and then transported to growing roots and coleoptile [44,47]. In germinating seeds of peas, sucrose is released from quickly degraded raffinose family oligosaccharides (RFOs), i.e., raffinose, stachyose, and verbascose, present at elevated concentrations in both the embryonic axis and cotyledons of mature seeds. The hydrolysis of RFOs starts just during seed imbibition and finishes in the embryonic axis at the end of seed germination (ca. 28–32 h after the start of seed imbibition), whereas in cotyledons, this process undergoes up to 7–10 days of germination [48,49]. Importantly, both products of RFOs hydrolysis, galactose and sucrose, are necessary for proper seedling growth, as documented in pea [50] and winter vetch [51]. Sucrose in sink tissues is hydrolyzed by sucrose synthase (Sus) and/or invertase (INV) to glucose and fructose—both serve as substrates for respiration and a source of carbon skeletons for other metabolic pathways [47].

Sucrose accumulation in roots and epicotyl/coleoptile under ZnO NPs treatment, found in our study (Figure 5), might be related to the inhibition of Sus and/or INV activity [41]. The increased activity of sucrose-phosphate synthase (SPS), an enzyme resynthesizing sucrose from monosaccharides, is also possible [47]. Although small-sized zinc oxide nanoparticles (ZnO NPs)—pyramids, plates, and spheres—possess the ability to inhibit the activity of a typical enzyme β-galactosidase in a biomimetic way [52], our results exclude a profound effect of ZnO NPs on α-galactosidase activity—the concentration of raffinose and stachyose in cotyledons (in 4-day-old seedlings they were absent) under ZnO NPs treatment was as low as in the control (Table S7). Thus, an excess of ZnO NPs seems to not disturb RFOs degradation during pea seed germination and seedling growth.

It could be expected that changes in sucrose and monosaccharides (fructose and glucose) would be related. Only in pea cv. Tarchalska (in both roots and epicotyls) and wheat cv. Ostka Strzelecka (in roots), a negative correlation between sucrose and monosaccharides level was observed (cv. Tarchalska *r* ≥ −0.86, cv. Ostka Strzelecka *r* = −0.97; Tables S11 and S12). However, such an effect caused by ZnO NPs was not uniform—the mentioned changes were not found in pea seedlings of cv. Nemo (Table S11) and in wheat coleoptiles (both cultivars; Tables S8 and S9). Moreover, in the roots of cv. Collada, the accumulation of sucrose was accompanied by the accumulation of monosaccharides (Table S8).

The seed germination and fast rate of early seedlings’ growth are accompanied by intensive respiration [43,48]. Thus, the changes in tricarboxylic acids observed in growing seedlings under ZnO NPs treatment, as found in our study in roots of peas (Figure 4) and wheat (Tables S8 and S9), can be a result of the impact on the TCA cycle [30]. A decrease in malic acid content in wheat roots was revealed earlier, under Ag NPs treatment [38] and in cucumber roots after foliar spray with ZnO NPs [53]. On the other hand, the accumulation of organic acids, such as malate and citrate (as in peas, Figure 4), is linked with heavy-metal tolerance [54,55,56]. They act as metal chelators, bind them in the cell cytosol, and immobilize in vacuoles to maintain their non-toxic level but also play an important role in metals transport through the xylem [54,56,57]. Moreover, organic acids can increase the expression of heavy-metal transporters and antioxidative enzymes [56]. Citric and malic acids are the main ligands of zinc in the xylem, participate in transport, and are involved in zinc sequestration in vacuoles, especially malate [55,56]. Therefore, malate and citrate accumulation in pea seedlings might contribute to their less noticeable sensitivity to ZnO NPs than in wheat seedlings.

Pea and wheat seedlings differ also in the case of changes in the concentration of amino acids caused by ZnO NPs exposure. In pea seedlings, homoserine (a predominant amino acid) decreased after ZnO NPs treatment, but it was more pronounced in epicotyls (Figure 4B, Tables S5 and S6). Homoserine takes part in the mobilization and transport of storage reserves from seeds and is synthesized in pea seedlings after germination [58]. Homoserine is synthesized from aspartate in a series of reactions catalyzed by aspartate kinase, aspartate semialdehyde dehydrogenase, and homoserine dehydrogenase [59]. Homoserine content decreased after ZnO NPs treatment, whereas aspartate increased (Tables S5 and S6). This suggests the inhibition of homoserine synthesis, which might be related to the inhibitory activity of ZnO NPs against dehydrogenase [30]. Regarding an accumulation of aspartic acid, glutamic acid, β-alanine, and GABA in pea roots and epicotyls (Tables S5 and S6), it could be suggested that ZnO NPs affect the aspartate–glutamate pathway. The above-mentioned proteinogenic (aspartate, glutamate) and non-proteinogenic (β-alanine, GABA) amino acids are important for the proper functioning of plants, and all of them were reported to accumulate in response to various abiotic stresses [60,61,62,63,64]. Glutamate and aspartate accumulation was also observed in tomato roots and leaves after foliar spray with ZnO NPs [46]. The accumulation of glutamate, aspartate, and β-alanine in peas, which are involved in the TCA cycle, might be also related to the accumulation of malate and citrate.

In wheat, ZnO NPs caused a decrease in the concentration of total amino acids (Figure 5), including asparagine and valine (in roots and coleoptile), glutamine (in roots only), and isoleucine (in coleoptile). ZnO NPs treatment increased the mobilization of amino acids from the endosperm, but their amount also decreased in seedlings, which suggests an increased nitrogen requirement and consumption. Asparagine can be synthesized from glutamine, and both are considered transport and storage molecules of reduced nitrogen in plants [65]. Isoleucine and valine, branched-chain amino acids, provide energy during germination and the later phase of seedling development [66].

GABA accumulation in the roots of both wheat and peas was a common response of both species to ZnO NPs. Accumulation of this amino acid has also been observed in pea roots after treatment with Ag NPs [67] and in maize leaves after growth in soil with SiO_2_, TiO_2_, and Fe_3_O_4_ engineered nanomaterials [68]. GABA is a signaling molecule, osmolyte, and also enhances the activity of antioxidant enzymes, important in plants’ response to various stresses [61]. Plants’ reaction to ZnO NPs might be related to heavy-metal stress, including zinc, which is associated with proline accumulation [69]. However, in our study, changes in proline levels were not related to ZnO NPs treatment.

## 4. Materials and Methods

### 4.1. Plant Material

Pea (*Pisum sativum* L.) seeds cultivars Nemo and Tarchalska, purchased from Danko Hodowla Roślin (Choryń, Poland) and spring wheat (*Triticum aestivum* L.) grains cultivars KWS Collada and Ostka Strzelecka, purchased from KWS and Hodowla Roślin Strzelce (Strzelce, Poland), respectively, were used in the experiment.

### 4.2. Preparation of ZnO NPs Suspension

ZnO NPs with a diameter size < 50 nm (cat. no. 677450-5G, Sigma-Aldrich, St. Louis, MO, USA) and <100 nm (cat. no. 721077-100G, Sigma-Aldrich, St. Louis, MO, USA) were used in the experiments. Nanoparticles were suspended in the double-distilled water by sonication 2 times for 30 min (Sonic-3, 310 W, 40 KHz, POLSONIC Pałczyński, Warszawa, Poland) to obtain ZnO NPs suspensions at specific concentrations for further experiments. 

### 4.3. Preliminary Study

In the preliminary study, the phytotoxic concentrations of ZnO NPs for seed germination and early seedling development of pea and wheat were selected. In the first experiment, pea seeds (cv. Nemo and cv. Tarchalska) and wheat grains (cv. Collada and cv. Ostka Strzelecka) were incubated in petri dishes (ø 12 cm) in water and ZnO NPs (diameter <50 nm; cat. no. 677450-5G, Sigma-Aldrich) suspensions at concentrations 20, 50, 250, 500, and 1000 mg/L for 4 and 3 days (pea and wheat, respectively) at 22 °C in the dark. After incubation, germination rate, seedlings’ length, and fresh and dry weight (FW and DW) were measured.

In the second experiment, pea seeds (cv. Tarchalska) and wheat grains (cv. Ostka Strzelecka) were incubated in petri dishes (ø 12 cm) in water and ZnO NPs of diameter <50 nm (cat. no. 677450-5G, Sigma-Aldrich) and <100 nm (cat. no. 721077-100G, Sigma-Aldrich) suspensions at concentrations 100, 250, and 1000 mg/L for 4 and 3 days (pea and wheat, respectively) at 22 °C in the dark. After incubation, germination rate, seedlings’ length, FW, and DW were measured.

### 4.4. The Effect of ZnO NPs on Seed Germination and Seedling Development

Pea seeds (cv. Nemo and cv. Tarchalska) and wheat grains (cv. Collada and cv. Ostka Strzelecka) were incubated in petri dishes (ø 12 cm) containing water and ZnO NPs (diameter <50 nm) suspensions at concentrations 100, 250, and 1000 mg/L for 4 and 3 days (pea and wheat, respectively) at 22 °C in the dark. The germinability of seeds was monitored daily. After incubation, the seedlings’ length and fresh and dry weight (FW and DW) were measured. Next, the tissues of pea seedlings (root, epicotyl, and cotyledons) and wheat seedlings (roots with scutellum, coleoptile, and endosperm) were frozen in liquid nitrogen and stored at −80 °C for the polar metabolite extraction.

### 4.5. Polar Metabolites Analyzes

Tissue samples of pea (root, epicotyl, and cotyledons) and wheat (roots, coleoptile, and endosperm) seedlings were freeze-dried and pulverized. The extraction was performed according to Szablińska-Piernik and Lahuta [70]. The polar metabolites were extracted with a mixture of methanol:water (1:1, *v*/*v*) at 70 °C for 30 min with continuous shaking. After cooling and centrifugation, cold chloroform was used to remove the non-polar compounds. The samples were dried and derivatized in two steps—with O-methoxamine hydrochloride and with a mixture of N-methyl-N-trimethylsilyl-trifluoroacetamide (MSTFA) and pyridine (1:1, *v*/*v*). The mixtures of trimethylsilyl (TMS)-derivatives were separated on a ZEBRON ZB-5MSi Guardian capillary column (Phenomenex, Torrance, CA, USA) in the gas chromatograph GC2010 Nexia (Shimadzu, Japan) with the flame ionization detector (FID). To confirm accurate metabolite identification, the gas chromatograph coupled with a quadrupole mass spectrometer (QP-GC-2010, Shimadzu, Japan) was used. Metabolites were identified and characterized by comparison of their retention time (RT), retention indices (RI, determined according to the saturated hydrocarbons), and mass spectra of original standards derived from Sigma-Aldrich (Sigma-Aldrich, Merck, Burlington, MA, USA) and from the NIST library (National Institute of Standards and Technology), as previously described [40,70].

### 4.6. Statistics

The results are the mean of 3 independent replicates and were subjected to one-way ANOVA with a post hoc test (Tukey, if overall *p* < 0.05) using the Statistica software (version 12.0; StatSoft, Tulsa, OK, USA). Graphs were prepared using GraphPad Prism (version 8; GraphPad Software, San Diego, CA, USA). Principal component analysis (PCA) for multivariate statistics was performed using COVAIN [71], a MATLAB toolbox including a graphical user interface (MATLAB version 2013a; Math Works, Natick, MA, USA). Additionally, Pearson’s correlation was performed for selected metabolites using Statistica software (version 12.0; StatSoft, Tulsa, OK, USA). Calculated Pearson correlation coefficients are presented in Tables S11 and S12 in Supplementary Materials.

## 5. Conclusions

The obtained results revealed species-, cultivar-, and organ-specific changes in the physiology and polar metabolite profiles of pea and wheat seedlings treated with ZnO NPs. Nanoparticles did not affect pea and wheat seeds’ germination. However, harmful effects on seedling growth, especially roots, were observed at all tested concentrations. Inhibition of root elongation was observed, particularly noticeable in wheat seedlings—reduction of radicle length over 50% at 100 mg/L ZnO NPs. Shoots were less affected, which might be related to the differences in the transport of ZnO NPs/Zn^2+^ ions in seedlings. This effect on seedlings’ growth could be a result of the over-uptake of ZnO NPs by the roots, ROS accumulation, and disturbance in phytohormone homeostasis. Our findings suggest that zinc oxide nanoparticles can affect sucrose metabolism in seedlings’ roots and cause oxidative stress, as indicated by the common reaction in pea and wheat roots—accumulation of sucrose and GABA.

Pea and wheat differ in their sensitivity to ZnO NPs, which may be related to differences in changes in the metabolomic profiles caused by nanoparticles. In wheat, ZnO NPs disrupted sugar mobilization in endosperms, probably by inhibiting α-amylase activity, as indicated by the decreasing content of maltose and maltotriose in a dose-dependent manner. Moreover, in wheat roots, malate decreased, and in seedlings and endosperms, the total amino acids content decreased, which indicates a disturbance in the TCA cycle and increased nitrogen requirement and consumption. In peas, the mobilization of storage materials in cotyledons was uninterrupted, but in seedlings, the accumulation of citric acid, malic acid, glutamate, aspartate, and β-alanine in roots, and citric and malic acid in epicotyls cv. Nemo, was observed. Therefore, the accumulation of such metabolites, which indicates changes in the aspartate–glutamate pathway and the TCA cycle, might contribute to the less noticeable sensitivity of pea seedlings to ZnO NPs than wheat seedlings. Further studies are required to verify this hypothesis. Our findings confirm plant species-specific reactions to ZnO NPs, which should be considered in potential agricultural applications. Using nanoparticles for seed nano-priming seems to be an optimistic solution—shorter exposure of seeds to ZnO NPs (for a few hours instead of a few days) can eliminate the harmful effects of ZnO NPs (presumably even at the highest concentrations tested here) while maintaining their beneficial properties (nutritional and biocidal properties against phytopathogens).

## Figures and Tables

**Table 1:** The concentration of predominant polar metabolites in roots and epicotyl/coleoptile of 4-day-old control seedlings of pea (*Pisum sativum* L.) of cultivars Nemo (N) and Tarchalska (T) and 3-day-old seedlings of wheat (*Triticum aestivum* L.) of cultivars Collada (C) and Ostka Strzelecka (OS). Values (in mg/g DW) are means of 3 replicates.

Metabolite	*Pisum sativum* L.				*Triticum aestivum* L.			
	Root		Epicotyl		Roots		Coleoptile	
	N	T	N	T	C	OS	C	OS
Sucrose	34.61	21.68	28.22	27.81	21.36	23.31	2.10	12.97
Glucose	8.70	16.34	13.72	14.73	24.38	21.57	46.59	41.83
Galactose	3.23	5.79	5.38	6.69	13.46	4.25	7.94	9.18
Fructose	0.84	1.27	3.73	3.43	10.61	10.68	32.21	28.12
Asparagine	3.00	2.22	3.95	4.88	5.80	5.83	2.45	2.70
Citrate	3.32	2.29	4.47	3.98	5.61	k4.90	5.33	6.22
Malate	4.83	3.22	4.34	5.43	16.05	12.48	2.38	2.92
Homoserine	26.59	30.33	24.36	30.36	- *	-	-	-
1-Kestose	- *	-	-	-	4.02	4.63	1.84	7.49
Glutamine	0.11	0.10	0.07	0.08	4.26	3.87	0.48	0.70
Hydroxyproline	0.48	0.27	0.29	0.18	2.30	2.88	0.72	2.55
Phosphoric acid	10.28	6.79	9.36	9.01	6.87	7.74	5.49	6.37

- * not detected.

## Data Availability

Not applicable.

## Supplementary Materials

The supporting information can be downloaded at https://www.mdpi.com/article/10.3390/ijms241914992/s1.
