# Supplementary material for: Zinc Oxide Nanoparticles Affect Early Seedlings’ Growth and Polar Metabolite Profiles of Pea (Pisum sativum L.) and Wheat (Triticum aestivum L.)
Source: Int J Mol Sci. 2023 Oct 8;24(19):14992. doi: 10.3390/ijms241914992 (PMC10573449; doi:10.3390/ijms241914992)
Supplement: Supplementary file 1 [file ijms-24-14992-s001.zip › ijms-2638159-supplementary.pdf]

Table S1. The germinability of pea seeds, length of root and epicotyl, fresh and dry weight (FW and DW, respectively) of root, epicotyl and cotyledons of 4-day-old seedling of pea (*Pisum sativum* L.) cv. Nemo and cv. Tarchalska developed in water suspensions of ZnO NPs at a concentration of 0, 20, 50, 250, 500 and 1000 mg/L. Values are means of 3 replicates. The same letters by the values indicate no statistically significant ($p < 0.05$) differences (valid for roots, coleoptiles and endosperms separately, individually for each cultivar) based on ANOVA analysis and Tukey's post- hoc corrections.

		**cv. Nemo**						**cv. Tarchalska**					
		ZnO NPs, mg/L											
		0*	20	50	250	500	1000	0*	20	50	250	500	1000
**germinability (%)**		100^{a}	98.3^{a}	100^{a}	100^{a}	100^{a}	100^{a}	100^{a}	100^{a}	100^{a}	100	100^{a}	100^{a}
**length**	root	40.93^{a}	37.25^{a}	43.98^{a}	31.19^{a}	32.44^{a}	30.26^{a}	43.02^{a}	42.22^{a}	40.87^{a}	27.82^{b}	27.31^{b}	28.94^{b}
**(mm)**	epicotyl	17.86^{a}	13.34^{a}	19.59^{a}	8.44^{a}	16.22^{a}	11.15^{a}	23.59^{a}	17.18^{ab}	18.67^{ab}	9.71^{b}	11.54^{b}	14.13^{ab}
**FW**	root	65.98^{a}	57.87^{a}	62.26^{a}	46.92^{a}	54.38^{a}	47.02^{a}	83.87^{a}	71.20^{ab}	66.30^{ab}	52.13^{b}	54.74^{b}	69.50^{ab}
**(mg)**	epicotyl	73.85^{a}	60.85^{a}	82.09^{a}	52.11^{a}	63.88^{a}	52.81^{a}	110.05^{a}	82.08^{ab}	80.17^{ab}	59.44^{b}	63.28^{b}	67.41^{b}
**DW**	root	4.68^{a}	4.16^{a}	4.54^{a}	3.78^{a}	4.42^{a}	4.44^{a}	6.34^{a}	5.57^{ab}	5.42^{abc}	3.93^{c}	4.54^{bc}	5.99^{ab}
**(mg)**	epicotyl	6.09^{a}	5.32^{a}	7.02^{a}	4.14^{a}	5.68^{a}	4.00^{a}	8.86	6.92^{a}	6.87^{ab}	5.36^{ab}	5.52^{b}	5.89^{b}

*- double distilled water – control

Table S2. The germinability of wheat grains, length of radicle and coleoptile, fresh and dry weight (FW and DW, respectively) of roots, coleoptile and endosperm, and germinability of 3-day-old seedling of wheat (*Triticum aestivum* L.) cv. Collada and cv. Ostka Strzelecka developed in water suspensions of ZnO NPs at a concentration of 0, 20, 50, 250, 500 and 1000 mg/L. Values are means of 3 replicates. The same letters by the values indicate no statistically significant ($p < 0.05$) differences (valid for roots, coleoptiles and endosperms separately, individually for each cultivar) based on ANOVA analysis and Tukey's post- hoc corrections.

		**cv. Collada**						**cv. Ostka Strzelecka**					
		ZnO NPs, mg/L											
		0*	20	50	250	500	1000	0*	20	50	250	500	1000
**germinability (%)**		96.67^{a}	96.67^{a}	96.67^{a}	98.33^{a}	100.0^{a}	100.0^{a}	98.33^{a}	98.33^{a}	93.33^{a}	93.33^{a}	93.33^{a}	96.67^{a}
**length**	root**	36.82^{a}	36.65^{a}	27.96^{ab}	22.24^{bc}	19.22^{bc}	16.20^{c}	26.21^{a}	24.29^{a}	19.87^{ab}	22.30^{ab}	16.33^{bc}	12.50^{c}
**(mm)**	coleoptile	17.14^{a}	16.88^{a}	17.76^{a}	14.99^{a}	13.44^{a}	13.08^{a}	11.96^{a}	11.33^{a}	10.61^{a}	11.80^{a}	10.28^{a}	10.97^{a}
**FW**	roots	27.32^{abc}	33.36^{a}	31.40^{ab}	23.32^{bc}	23.02^{bc}	18.84^{c}	19.85^{a}	18.03^{ab}	16.38^{ab}	15.58^{abc}	13.68^{bc}	11.45^{c}
**(mg)**	coleoptile	21.77^{a}	19.82^{a}	22.08^{a}	19.53^{a}	16.88^{a}	16.98^{a}	16.15^{a}	13.61^{a}	14.06^{a}	15.45^{a}	14.68^{a}	14.89^{a}
**DW**	roots	2.62^{a}	2.85^{a}	2.78^{a}	2.41^{a}	2.26^{a}	2.04^{a}	1.65^{a}	1.53^{a}	1.93^{a}	1.56^{a}	1.52^{a}	1.45^{a}
**(mg)**	coleoptile	2.21^{a}	1.99^{a}	2.34^{a}	2.31^{a}	2.04^{a}	1.99^{a}	1.84^{a}	1.62^{a}	1.70^{a}	1.83^{a}	1.85^{a}	1.87^{a}

*- double distilled water – control; **- radicle

Table S3. The germinability of pea seeds, length of root and epicotyl, fresh and dry weight (FW and DW, respectively) of root, epicotyl and cotyledons of 4-day-old seedling of pea (*Pisum sativum* L.) cv. Tarchalska developed in water suspensions of ZnO NPs with a diameter <50 and <100 nm, at a concentration of 0, 100, 250 and 1000 mg/L. Values are means of 3 replicates. The same letters by the values indicate no statistically significant ($p < 0.05$) differences (valid for roots, coleoptiles and endosperms separately, individually for each nanoparticle size experiment) based on ANOVA analysis and Tukey's post- hoc corrections.

		**ZnO NPs diameter <50nm**				**ZnO NPs diameter <100nm**			
		ZnO NPs, mg/L							
		0*	100	250	1000	0*	100	250	100
**germinability (%)**		100^{a}	100^{a}	100^{a}	100^{a}	100^{a}	100^{a}	100^{a}	100^{a}
**length**	root	37.52^{a}	36.38^{a}	30.74^{a}	28.12^{a}	40.04^{a}	33.87^{a}	24.23^{b}	20.32^{b}
**(mm)**	epicotyl	20.72^{a}	9.53^{b}	12.63^{b}	13.28^{b}	17.80^{a}	14.30^{a}	12.71^{a}	15.02^{a}
**FW**	root	97.61^{a}	64.89^{b}	62.41^{b}	56.74^{b}	97.06^{a}	77.83^{ab}	69.02^{ab}	63.61^{b}
**(mg)**	epicotyl	98.74^{a}	71.95^{a}	76.82^{a}	85.55^{a}	70.11^{a}	64.68^{a}	57.20^{a}	59.13^{a}
**DW**	root	7.89^{a}	5.81^{b}	5.68^{b}	5.34^{b}	7.32^{a}	6.27^{a}	5.78^{a}	5.69^{a}
**(mg)**	epicotyl	8.34^{a}	6.67^{a}	7.11^{a}	7.83^{a}	5.62^{a}	5.60^{a}	5.05^{a}	5.19^{a}

*- double distilled water – control

Table S4. The germinability of wheat grains, length of root and coleoptile, fresh and dry weight (FW and DW, respectively) of roots, coleoptile and endosperm of 3-day-old seedling of wheat (*Triticum aestivum* L.) cv. Ostka Strzelecka developed in water suspensions of ZnO NPs with a diameter <50 and <100 nm, at a concentration of 0, 100, 250 and 1000 mg/L. Values are means of 3 replicates. The same letters by the values indicate no statistically significant ($p < 0.05$) differences (valid for roots, coleoptiles and endosperms separately, individually for each nanoparticle size experiment) based on ANOVA analysis and Tukey's post- hoc corrections.

		**ZnO NPs diameter < 50nm**				**ZnO NPs diameter < 100nm**			
		ZnO NPs, mg/L							
		0*	100	250	1000	0*	100	250	100
**germinability (%)**		95.83^{a}	97.50^{a}	94.17^{a}	95.83^{a}	94.17^{a}	95.00^{a}	96.67^{a}	90.83^{a}
**length**	root**	27.68^{a}	13.29^{b}	9.50^{bc}	7.91^{c}	27.34^{a}	13.61^{b}	11.26^{bc}	8.72^{c}
**(mm)**	coleoptile	12.88^{a}	12.54^{a}	11.70^{a}	10.61^{a}	12.69^{a}	11.47^{a}	10.95^{a}	10.92^{a}
**FW**	roots	21.04^{a}	15.64^{b}	13.80^{bc}	11.74^{c}	25.70^{a}	16.74^{b}	20.17^{ab}	15.88^{b}
**(mg)**	coleoptile	13.42^{a}	13.28^{a}	12.60^{ab}	11.08^{b}	14.08^{a}	11.48^{a}	12.95^{a}	12.41^{a}
**DW**	roots	1.94^{a}	2.03^{a}	1.92^{a}	1.65^{a}	2.07^{a}	1.81^{a}	2.13^{a}	1.94^{a}
**(mg)**	coleoptile	1.70^{a}	1.92^{a}	1.80^{a}	1.55^{a}	1.62^{a}	1.50^{a}	1.69^{a}	1.63^{a}

*- double distilled water – control; **- radicle

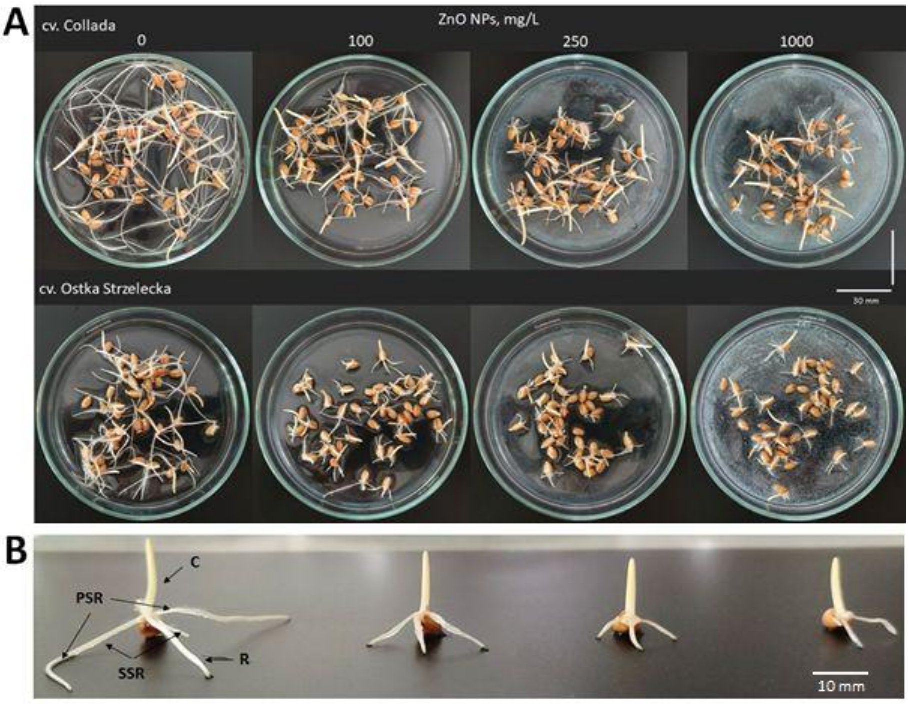

Figure S1. The inhibitory effects of ZnO NPs on the growth and development of wheat seedlings cv. Collada and cv. Ostka Strzelecka (A). The inhibition of seedlings growth (elongation of radicle and primary seminal roots) and restriction of the development of the second pair of seminal roots in wheat seedlings of cv. Ostka Strzelecka was shown on the bottom panel (B). *Abbreviations*: R – radicle, PSR – first pair of seminal roots, SSR – second pair of seminal roots, C – coleoptile.

Table S5. The concentration of total identified polar metabolites (**TIPMs**), including total soluble carbohydrates (**TSCs**), total amino acids (**TAAs**), total organic acids (**TOAs**), and total remaining compounds (**TRCs**) in root and epicotyl of 4-day-old seedlings of pea (*Pisum sativum* L.) cv. Nemo developed in water suspensions of ZnO NPs at a concentration of 0, 100, 250 and 1000 mg/L. Values (in mg/g DW) are means of 3 replicates. The same letters by the values indicate no statistically significant ($p < 0.05$) differences (valid for roots and epicotyls separately) based on ANOVA analysis and Tukey's post-hoc corrections.

Metabolites	**Root**				**Epicotyl**			
	ZnO NPs, mg/L							
	0*	100	250	1000	0*	100	250	1000
**TIPMs:**	**115.61[c]**	**126.97[ab]**	**133.91[a]**	**121.00[bc]**	**114.68[b]**	**119.78[b]**	**132.50[a]**	**134.00[a]**
**TSCs:**	**51.21[c]**	**58.97[b]**	**65.15[a]**	**53.96[c]**	**54.87[d]**	**64.47[c]**	**74.09[b]**	**82.02[a]**
fructose	0.84[a]	0.59[a]	0.42[ab]	0.10[b]	3.71[b]	4.34[a]	4.05[ab]	3.74[ab]
galactose	3.23[a]	2.44[b]	2.72[b]	2.34[b]	5.38[b]	2.95[c]	4.22[bc]	11.05[a]
glucose	8.70[a]	8.26[a]	8.95[a]	7.93[a]	13.72[b]	6.71[d]	9.52[c]	25.07[a]
myo-inositol	3.22[a]	3.04[a]	2.92[a]	3.06[a]	3.21[a]	3.57[a]	3.93[a]	3.36[a]
sucrose	34.61[c]	44.11[b]	49.70[a]	40.07[b]	28.22[d]	46.01[b]	51.74[a]	38.43[c]
erythronic acid	0.24[a]	0.21[a]	0.21[a]	0.24[a]	0.36[a]	0.25[ab]	0.34[a]	0.20[b]
gluconic acid	0.36[a]	0.32[a]	0.22[b]	0.21[b]	0.26[b]	0.64[a]	0.30[b]	0.17[b]
**TAAs:**	**44.14[a]**	**42.46[a]**	**41.90[a]**	**42.27[a]**	**39.90[a]**	**33.77[bc]**	**36.36[ab]**	**30.83[c]**
alanine	2.89[b]	3.77[a]	3.19[b]	1.67[c]	1.11[b]	2.77[a]	2.75[a]	1.21[b]
arginine	0.04[a]	0.11[a]	0.14[a]	0.05[a]	0.00[a]	0.04[a]	0.00[a]	0.00[a]
asparagine	3.00[a]	2.33[b]	3.19[a]	2.39[b]	3.95[a]	2.72[d]	3.15[c]	3.54[b]
aspartic acid	1.50[b]	1.76[a]	1.88[a]	1.65[ab]	1.03[b]	1.42[a]	1.39[a]	1.06[b]
β-alanine	0.30[b]	0.47[ab]	0.61[a]	0.57[a]	0.27[b]	0.46[a]	0.42[a]	0.33[b]
GABA	1.39[d]	2.08[c]	2.50[b]	2.96[a]	1.70[c]	2.98[a]	2.81[a]	2.29[b]
glutamic acid	1.55[a]	1.76[a]	1.92[a]	1.75[a]	0.95[a]	1.06[a]	1.07[a]	0.94[a]
glutamine	0.11[b]	0.14[a]	0.12[ab]	0.14[a]	0.07[a]	0.09[a]	0.08[a]	0.11[a]
homoserine	26.59[a]	22.44[a]	21.05[a]	25.15[a]	24.36[a]	14.98[b]	14.30[b]	15.96[b]
hydroxyproline	0.48[a]	0.59[a]	0.65[a]	0.52[a]	0.29[b]	0.35[ab]	0.45[a]	0.25[b]
isoleucine	0.72[a]	0.87[a]	0.91[a]	0.74[a]	0.69[a]	0.94[a]	1.31[a]	0.68[a]
leucine	0.03[a]	0.04[a]	0.04[a]	0.02[a]	0.03[b]	0.06[a]	0.05[a]	0.03[b]
lysine	0.70[a]	0.56[a]	0.45[a]	0.49[a]	0.00[c]	1.05[b]	2.59[a]	0.00[c]
phenylalanine	0.21[a]	0.18[a]	0.21[a]	0.13[b]	0.40[a]	0.36[a]	0.43[a]	0.38[a]
proline	1.08[bc]	1.69[a]	1.34[b]	1.02[c]	0.62[a]	1.20[a]	1.52[a]	0.61[a]
serine	1.78[a]	1.94[a]	1.87[a]	1.48[b]	1.10[bc]	1.27[ab]	1.33[a]	0.98[c]
threonine	0.57[a]	0.50[a]	0.56[a]	0.58[a]	1.80[a]	0.67[c]	0.85[c]	1.31[b]
tyrosine	0.00	0.00	0.00	0.00	0.00[b]	0.00[b]	0.37[a]	0.00[b]
valine	1.20[a]	1.23[a]	1.28[a]	0.96[b]	1.54[a]	1.38[a]	1.49[a]	1.16[b]
**TOAs:**	**9.83[c]**	**14.23[b]**	**16.55[a]**	**15.97[a]**	**10.55[b]**	**12.05[a]**	**12.54[a]**	**12.65[a]**
acetic acid	0.19[a]	0.19[a]	0.20[a]	0.19[a]	0.21[a]	0.19[a]	0.21[a]	0.19[a]
butyric acid	0.12[a]	0.10[ab]	0.08[b]	0.09[b]	0.13[a]	0.06[b]	0.07[b]	0.10[ab]
citric acid	3.32[d]	4.12[c]	5.32[b]	6.07[a]	4.47[c]	4.56[c]	5.26[b]	6.28[a]
fumaric acid	0.15[a]	0.18[a]	0.17[a]	0.18[a]	0.21[a]	0.25[a]	0.21[a]	0.19[a]
glutaric acid	0.04[a]	0.04[a]	0.04[a]	0.05[a]	0.05[a]	0.03[a]	0.04[a]	0.05[a]
lactic acid	0.26[a]	0.19[b]	0.29[a]	0.16[b]	0.18[b]	0.27[a]	0.24[a]	0.14[b]
malic acid	4.83[c]	8.24[b]	9.39[a]	8.32[b]	4.34[c]	5.26[ab]	5.45[a]	4.74[bc]
malonic acid	0.10[c]	0.14[a]	0.13[ab]	0.11[bc]	0.05[c]	0.07[b]	0.08[a]	0.05[c]
oxalic acid	0.16[a]	0.14[a]	0.16[a]	0.16[a]	0.18[a]	0.13[b]	0.15[ab]	0.17[ab]
propionic acid	0.15[a]	0.16[a]	0.15[a]	0.14[a]	0.25[a]	0.62[a]	0.19[a]	0.22[a]
succinic acid	0.51[b]	0.74[a]	0.62[ab]	0.50[b]	0.47[c]	0.61[ab]	0.65[a]	0.51[bc]
**TRCs, including:**	**10.43[a]**	**11.30[a]**	**10.31[a]**	**8.79[b]**	**9.36[a]**	**9.49[a]**	**9.51[a]**	**8.50[a]**
phosphoric acid	10.28[a]	11.17[a]	10.18[b]	8.69[b]	9.29[a]	9.44[a]	9.45[a]	8.46[a]
urea	0.14[a]	0.14[a]	0.12[a]	0.10[a]	0.07[a]	0.05[a]	0.05[a]	0.05[a]

*- double distilled water – control

Table S6. The concentration of total polar metabolites (**TPMs**), including total soluble carbohydrates (**TSCs**), total amino acids (**TAAs**), total organic acids (**TOAs**), and total remaining compounds (**TRCs**) in root and epicotyl of 4-day-old seedlings of pea (*Pisum sativum* L.) cv. Tarchalska developed in water suspensions of ZnO NPs at a concentration of 0, 100, 250 and 1000 mg/L. Values (in mg/g DW) are means of 3 replicates. The same letters by the values indicate no statistically significant ($p < 0.05$) differences (valid for roots and epicotyls separately) based on ANOVA analysis and Tukey's post-hoc corrections.

Metabolites	**Root**				**Epicotyl**			
	ZnO NPs, mg/L							
	0*	100	250	1000	0*	100	250	1000
**TIPMs:**	**115.47^{bc}**	**101.49^{c}**	**114.50^{a}**	**111.03^{ab}**	**122.71^{a}**	**116.38^{ab}**	**110.67^{b}**	**115.46^{ab}**
**TSCs:**	**48.37^{a}**	**39.72^{b}**	**48.69^{a}**	**47.75^{a}**	**56.42^{a}**	**58.56^{a}**	**56.44^{a}**	**60.93^{a}**
fructose	1.27^{a}	0.14^{c}	0.22^{b}	0.09^{c}	3.43^{a}	2.31^{b}	2.21^{b}	2.31^{b}
galactose	5.79^{a}	1.24^{b}	1.26^{b}	1.19^{b}	6.69^{a}	1.71^{c}	4.34^{b}	5.27^{ab}
glucose	16.34^{a}	4.53^{b}	4.36^{b}	4.88^{b}	14.73^{a}	4.38^{c}	7.62^{b}	10.52^{b}
myo-inositol	2.86^{a}	2.06^{b}	1.90^{b}	2.24^{b}	3.26^{a}	2.85^{a}	2.90^{a}	2.82^{a}
sucrose	21.68^{c}	31.36^{b}	40.63^{a}	38.98^{a}	27.81^{c}	46.94^{a}	38.96^{b}	39.62^{b}
erythronic acid	0.19^{b}	0.22^{a}	0.20^{ab}	0.22^{a}	0.28^{a}	0.24^{b}	0.27^{a}	0.28^{a}
gluconic acid	0.24^{a}	0.16^{b}	0.13^{b}	0.16^{b}	0.21^{a}	0.14^{b}	0.14^{b}	0.12^{b}
**TAAs:**	**42.13^{a}**	**41.83^{a}**	**43.02^{a}**	**41.29^{a}**	**46.14^{a}**	**41.13^{b}**	**37.22^{bc}**	**36.40^{c}**
alanine	0.94^{c}	1.72^{a}	1.21^{b}	0.84^{c}	0.92^{c}	2.26^{a}	1.51^{b}	0.91^{c}
arginine	0.00^{c}	0.13^{a}	0.06^{b}	0.05^{bc}	0.01^{b}	0.03^{a}	0.00^{b}	0.00^{b}
asparagine	2.22^{b}	2.48^{a}	2.26^{b}	2.55^{a}	4.88^{a}	2.84^{b}	2.53^{b}	2.91^{b}
aspartic acid	1.00^{b}	1.39^{a}	1.32^{a}	1.30^{a}	0.97^{b}	1.60^{a}	1.13^{b}	1.01^{b}
β-alanine	0.35^{c}	0.53^{b}	0.58^{a}	0.51^{b}	0.21^{c}	0.56^{a}	0.36^{b}	0.19^{c}
GABA	1.41^{c}	1.71^{b}	2.12^{a}	2.24^{a}	1.79^{c}	2.47^{a}	2.08^{b}	1.67^{c}
glutamic acid	0.76^{b}	1.46^{a}	1.51^{a}	1.41^{a}	0.76^{a}	1.15^{a}	1.04^{c}	0.89^{b}
glutamine	0.10^{a}	0.11^{a}	0.14^{a}	0.13^{a}	0.08^{a}	0.09^{a}	0.06^{b}	0.06^{b}
homoserine	30.33^{a}	26.39^{b}	28.77^{ab}	27.99^{ab}	30.36^{a}	23.78^{b}	21.54^{b}	22.21^{b}
hydroxyproline	0.27^{b}	0.50^{a}	0.52^{a}	0.46^{a}	0.18^{b}	0.45^{a}	0.33^{a}	0.31^{ab}
isoleucine	0.49^{c}	0.71^{a}	0.61^{b}	0.57^{b}	0.55^{c}	0.81^{a}	0.71^{ab}	0.63^{bc}
leucine	0.02^{ab}	0.03^{ab}	0.03^{a}	0.02^{b}	0.03^{a}	0.03^{a}	0.03^{a}	0.02^{a}
lysine	0.54^{ab}	0.88^{a}	0.39^{b}	0.33^{b}	0.00^{c}	0.48^{b}	0.77^{a}	1.23^{a}
phenylalanine	0.19^{a}	0.14^{ab}	0.17^{ab}	0.12^{b}	0.36^{b}	0.34^{b}	0.42^{a}	0.22^{c}
proline	0.39^{b}	0.59^{a}	0.60^{a}	0.41^{b}	0.37^{b}	0.53^{a}	0.52^{a}	0.33^{b}
serine	1.55^{a}	1.46^{a}	1.17^{b}	1.05^{b}	1.31^{b}	1.54^{a}	1.43^{ab}	1.18^{c}
threonine	0.55^{a}	0.46^{b}	0.49^{ab}	0.51^{ab}	2.03^{a}	0.75^{c}	1.35^{b}	1.49^{b}
tyrosine	0.00	0.00	0.00	0.00	0.00	0.00	0.00	0.00
valine	1.00^{b}	1.16^{a}	1.07^{b}	0.80^{c}	1.31^{a}	1.42^{a}	1.40^{a}	1.15^{b}
**TOAs:**	**7.07^{d}**	**10.26^{c}**	**13.60^{a}**	**12.93^{b}**	**11.15^{a}**	**9.90^{c}**	**10.16^{bc}**	**10.89^{ab}**
acetic acid	0.17^{a}	0.15^{b}	0.15^{b}	0.15^{b}	0.21^{a}	0.17^{ab}	0.15^{b}	0.18^{ab}
butyric acid	0.13^{a}	0.08^{c}	0.09^{c}	0.11^{b}	0.13^{a}	0.06^{c}	0.06^{c}	0.08^{b}
citric acid	2.29^{c}	1.82^{d}	2.76^{b}	3.10^{a}	3.98^{a}	3.09^{c}	3.52^{b}	3.97^{a}
fumaric acid	0.11^{b}	0.12^{b}	0.13^{b}	0.15^{a}	0.20^{a}	0.14^{b}	0.15^{b}	0.15^{b}
glutaric acid	0.03^{ab}	0.03^{b}	0.04^{ab}	0.04^{a}	0.06^{ab}	0.03^{c}	0.07^{a}	0.05^{b}
lactic acid	0.31^{a}	0.24^{b}	0.23^{bc}	0.21^{c}	0.19^{a}	0.17^{ab}	0.11^{c}	0.14^{bc}
malic acid	3.33^{d}	7.08^{c}	9.52^{a}	8.54^{b}	5.43^{a}	5.44^{a}	5.39^{a}	5.61^{a}
malonic acid	0.05^{b}	0.08^{a}	0.07^{a}	0.08^{a}	0.04^{c}	0.10^{a}	0.05^{b}	0.04^{c}
oxalic acid	0.12^{a}	0.10^{ab}	0.10^{b}	0.10^{b}	0.16^{a}	0.09^{b}	0.10^{b}	0.12^{b}
propionic acid	0.17^{a}	0.08^{b}	0.08^{b}	0.10^{b}	0.24^{a}	0.11^{d}	0.14^{c}	0.17^{b}
succinic acid	0.36^{c}	0.48^{a}	0.44^{b}	0.35^{c}	0.52^{a}	0.51^{a}	0.42^{b}	0.38^{b}
**TRCs:**	**6.79^{ab}**	**7.23^{a}**	**6.61^{b}**	**6.38^{b}**	**9.01^{a}**	**6.80^{b}**	**6.86^{b}**	**7.24^{b}**
phosphoric acid	6.71^{ab}	7.16^{a}	6.53^{b}	6.28^{b}	8.94^{a}	6.76^{b}	6.80^{b}	7.16^{b}
urea	0.08^{b}	0.08^{b}	0.09^{ab}	0.10^{a}	0.07^{ab}	0.04^{c}	0.05^{bc}	0.08^{a}

*- double distilled water - control

Table S7. The concentration of total identified polar metabolites (**TIPMs**), including total soluble carbohydrates (**TSCs**), total amino acids (**TAAs**), total organic acids (**TOAs**), and total remaining compounds (**TRCs**) in **cotyledons** of 4-day-old seedlings of pea (*Pisum sativum* L.) cv. Nemo and cv. Tarchalska developed in water suspensions of ZnO NPs at a concentration of 0, 100, 250 and 1000 mg/L. Values (in mg/g DW) are means of 3 replicates. The same letters by the values indicate no statistically significant ($p < 0.05$) differences (valid for cultivars separately) based on ANOVA analysis and Tukey's post- hoc corrections.

Metabolites	**cv. Nemo**				**cv. Tarchalska**			
	ZnO NPs, mg/L							
	0*	100	250	1000	0*	100	250	1000
**TIPMs**:	**59.79^{a}**	**58.73^{a}**	**57.42^{a}**	**57.96^{a}**	**57.57^{a}**	**55.66^{a}**	**58.45^{a}**	**57.53^{a}**
**TSCs**:	**46.29^{a}**	**48.13^{a}**	**46.31^{a}**	**44.07^{a}**	**40.82^{a}**	**43.53^{a}**	**45.53^{a}**	**42.79^{a}**
fructose	0.04^{a}	0.03^{ab}	0.03^{b}	0.03^{b}	0.05^{a}	0.06^{a}	0.06^{a}	0.05^{a}
galactose	0.14^{a}	0.07^{a}	0.07^{a}	0.05^{a}	0.05^{a}	0.06^{a}	0.06^{a}	0.06^{a}
glucose	0.25^{a}	0.18^{a}	0.18^{a}	0.15^{a}	0.15^{a}	0.13^{a}	0.16^{a}	0.16^{a}
myo-inositol	2.51^{a}	2.42^{a}	2.24^{a}	2.12^{a}	2.03^{a}	1.77^{a}	1.89^{a}	1.86^{a}
raffinose	0.63^{a}	0.64^{a}	0.67^{a}	0.34^{a}	0.32^{a}	0.47^{a}	0.52^{a}	0.61^{a}
sucrose	41.62^{a}	43.84^{a}	42.36^{a}	40.82^{a}	37.73^{a}	40.37^{a}	42.09^{a}	39.30^{a}
stachyose	1.11^{a}	0.94^{a}	0.76^{a}	0.55^{a}	0.50^{a}	0.68^{a}	0.77^{a}	0.75^{a}
**TAAs**:	**8.70^{a}**	**6.32^{a}**	**6.65^{a}**	**8.64^{a}**	**11.18^{a}**	**7.13^{b}**	**7.90^{b}**	**9.14^{ab}**
alanine	0.48^{a}	0.51^{a}	0.49^{a}	0.40^{a}	0.48^{a}	0.39^{a}	0.56^{a}	0.51^{a}
arginine	0.00^{b}	0.00^{b}	0.00^{ab}	0.01^{a}	0.20^{a}	0.15^{a}	0.14^{a}	0.19^{a}
asparagine	0.31^{a}	0.25^{a}	0.34^{a}	0.29^{a}	0.45^{a}	0.32^{b}	0.33^{b}	0.35^{b}
β-alanine	0.12^{a}	0.09^{a}	0.10^{a}	0.13^{a}	0.13^{a}	0.11^{a}	0.11^{a}	0.12^{a}
GABA	2.29^{a}	1.69^{a}	1.58^{a}	2.14^{a}	2.12^{a}	1.52^{a}	1.55^{a}	1.87^{a}
glutamic acid	0.60^{a}	0.48^{a}	0.56^{a}	0.66^{a}	0.91^{a}	0.50^{a}	0.72^{a}	0.70^{a}
glutamine	0.13^{a}	0.14^{a}	0.12^{a}	0.07^{a}	0.07^{a}	0.07^{a}	0.06^{a}	0.05^{a}
homoserine	1.26^{ab}	0.74^{b}	0.71^{b}	1.68^{a}	2.72^{a}	1.49^{a}	1.45^{a}	2.20^{a}
hydroxyproline	0.68^{a}	0.51^{a}	0.57^{a}	0.68^{a}	0.92^{a}	0.68^{a}	0.70^{a}	0.72^{a}
isoleucine	0.25^{a}	0.18^{b}	0.22^{ab}	0.21^{ab}	0.25^{a}	0.21^{b}	0.23^{ab}	0.22^{ab}
lysine	0.04^{a}	0.04^{a}	0.03^{a}	0.04^{a}	0.04^{b}	0.04^{b}	0.04^{b}	0.08^{a}
methionine	0.02^{a}	0.02^{a}	0.02^{a}	0.02^{a}	0.03^{a}	0.01^{a}	0.02^{a}	0.03^{a}
phenylalanine	0.25^{a}	0.12^{b}	0.16^{ab}	0.23^{a}	0.31^{a}	0.14^{b}	0.19^{b}	0.21^{ab}
proline	0.65^{a}	0.48^{a}	0.49^{a}	0.60^{a}	0.57^{a}	0.38^{b}	0.45^{ab}	0.47^{ab}
serine	0.94^{a}	0.62^{b}	0.72^{ab}	0.86^{ab}	1.18^{a}	0.61^{b}	0.78^{ab}	0.82^{ab}
threonine	0.07^{a}	0.05^{a}	0.07^{a}	0.07^{a}	0.12^{a}	0.08^{b}	0.08^{b}	0.08^{ab}
tyrosine	0.01^{b}	0.02^{ab}	0.03^{a}	0.02^{a}	0.02^{a}	0.02^{a}	0.02^{a}	0.02^{a}
valine	0.59^{a}	0.40^{b}	0.44^{ab}	0.52^{ab}	0.65^{a}	0.40^{b}	0.47^{b}	0.50^{ab}
**TOAs**:	**2.91^{ab}**	**2.63^{b}**	**2.80^{b}**	**3.54^{a}**	**3.52^{ab}**	**3.32^{b}**	**3.42^{ab}**	**4.05^{a}**
citric acid	1.51^{ab}	1.33^{b}	1.62^{ab}	2.06^{a}	2.17^{a}	2.12^{a}	2.32^{a}	2.85^{a}
fumaric acid	0.02^{a}	0.02^{a}	0.02^{a}	0.02^{a}	0.02^{a}	0.02^{a}	0.02^{a}	0.02^{a}
lactic acid	0.46^{a}	0.42^{a}	0.33^{a}	0.57^{a}	0.41^{a}	0.35^{a}	0.25^{a}	0.32^{a}
malic acid	0.24^{a}	0.27^{a}	0.28^{a}	0.28^{a}	0.29^{a}	0.32^{a}	0.35^{a}	0.36^{a}
malonic acid	0.10^{b}	0.11^{b}	0.13^{a}	0.10^{b}	0.05^{a}	0.07^{a}	0.06^{a}	0.05^{a}
propionic acid	0.05^{a}	0.04^{a}	0.04^{a}	0.05^{a}	0.04^{a}	0.03^{a}	0.03^{a}	0.04^{a}
succinic acid	0.51^{a}	0.43^{a}	0.39^{a}	0.46^{a}	0.53^{a}	0.40^{a}	0.40^{a}	0.40^{a}
**TRCs**:	**1.89^{a}**	**1.66^{a}**	**1.66^{a}**	**1.72^{a}**	**2.05^{a}**	**1.69^{ab}**	**1.60^{ab}**	**1.55^{b}**
phosphoric acid	1.87^{a}	1.65^{a}	1.64^{a}	1.70^{a}	2.03^{a}	1.67^{ab}	1.58^{ab}	1.53^{b}
urea	0.02^{a}	0.01^{a}	0.01^{a}	0.02^{a}	0.02^{a}	0.02^{a}	0.01^{a}	0.02^{a}

*- double distilled water - control

Table S8. The concentration of total identified polar metabolites (**TIPMs**), including total soluble carbohydrates (**TSCs**), total amino acids (**TAAs**), total organic acids (**TOAs**), and total remaining compounds (**TRCs**) in roots and coleoptile of 3-day-old seedlings of wheat (*Triticum aestivum* L.) cv. Collada developed in water suspensions of ZnO NPs at a concentration of 0, 100, 250 and 1000 mg/L. Values (in mg/g DW) are means of 3 replicates. The same letters by the values indicate no statistically significant ($p < 0.05$) differences (valid for roots and coleoptiles separately) based on ANOVA analysis and Tukey's post- hoc corrections.

Metabolites	**Roots**				**Coleoptile**			
	ZnO NPs, mg/L							
	0*	100	250	1000	0*	100	250	1000
**TIPMs:**	**131.33^{c}**	**131.28^{c}**	**144.69^{b}**	**167.36^{a}**	**115.39^{b}**	**126.25^{a}**	**127.46^{a}**	**109.93^{b}**
**TSCs**:	**78.85^{c}**	**85.96^{c}**	**102.38^{b}**	**128.84^{a}**	**92.05^{b}**	**103.87^{a}**	**106.67^{a}**	**89.23^{b}**
fructose	10.61^{b}	13.45^{b}	15.52^{ab}	20.37^{a}	32.21^{b}	37.82^{a}	38.06^{a}	30.15^{b}
galactose	13.46^{a}	11.94^{ab}	8.48^{b}	3.68^{c}	7.94^{a}	7.40^{ab}	7.06^{b}	6.82^{b}
glucose	24.38^{ab}	17.38^{b}	21.14^{b}	31.34^{a}	46.59^{bc}	50.30^{ab}	52.71^{a}	42.84^{c}
1-kestose	4.02^{d}	6.66^{c}	7.69^{b}	13.26^{a}	1.84^{b}	3.51^{a}	3.62^{a}	3.65^{a}
maltose	4.74^{b}	3.70^{b}	7.67^{a}	3.27^{b}	0.55^{a}	0.32^{bc}	0.34^{b}	0.30^{c}
myo-inositol	0.28^{a}	0.30^{a}	0.28^{a}	0.31^{a}	0.81^{a}	0.89^{a}	0.86^{a}	0.84^{a}
sucrose	21.36^{d}	32.53^{c}	41.60^{b}	56.61^{a}	2.10^{b}	3.64^{ab}	4.03^{ab}	4.65^{a}
**TAAs**:	**21.90^{a}**	**16.87^{c}**	**17.14^{c}**	**19.79^{b}**	**9.45^{a}**	**8.84^{ab}**	**8.13^{b}**	**7.85^{b}**
alanine	0.38^{c}	0.48^{b}	0.54^{b}	0.67^{a}	0.23^{a}	0.28^{a}	0.26^{a}	0.25^{a}
asparagine	5.80^{a}	3.81^{b}	3.54^{b}	3.14^{c}	2.45^{a}	1.63^{b}	1.63^{b}	1.68^{b}
aspartic acid	0.32^{a}	0.46^{a}	0.49^{a}	0.45^{a}	0.33^{a}	0.32^{ab}	0.30^{b}	0.30^{ab}
GABA	0.34^{b}	0.41^{b}	0.52^{a}	0.57^{a}	1.01^{b}	1.18^{a}	1.08^{b}	1.04^{b}
glutamic acid	1.09^{b}	1.45^{a}	1.36^{ab}	1.46^{a}	0.31^{a}	0.29^{ab}	0.28^{bc}	0.27^{c}
glutamine	4.26^{a}	2.78^{b}	2.59^{b}	2.72^{b}	0.48^{b}	0.52^{ab}	0.52^{ab}	0.56^{a}
glycine	0.46^{a}	0.40^{b}	0.37^{b}	0.37^{b}	0.51^{a}	0.50^{a}	0.47^{b}	0.40^{c}
hydroxyproline	2.30^{a}	1.69^{b}	1.63^{b}	2.17^{a}	0.72^{b}	0.80^{ab}	0.76^{ab}	0.94^{a}
isoleucine	0.71^{a}	0.49^{c}	0.50^{bc}	0.56^{b}	0.19^{a}	0.18^{a}	0.18^{ab}	0.16^{b}
leucine	0.65^{a}	0.46^{b}	0.45^{b}	0.46^{b}	0.10^{a}	0.11^{a}	0.11^{a}	0.09^{b}
lysine	1.69^{b}	1.07^{b}	1.35^{b}	2.77^{b}	0.99^{a}	0.91^{a}	0.55^{a}	0.31^{a}
methionine	0.03^{a}	0.02^{a}	0.03^{a}	0.03^{a}	0.01^{a}	0.01^{a}	0.01^{a}	0.01^{a}
phenylalanine	0.27^{b}	0.24^{b}	0.27^{b}	0.37^{a}	0.12^{a}	0.12^{a}	0.12^{a}	0.11^{a}
proline	1.25^{b}	1.17^{b}	1.26^{b}	1.64^{a}	0.20^{a}	0.20^{a}	0.18^{a}	0.29^{a}
serine	0.62^{b}	0.58^{c}	0.58^{c}	0.72^{c}	0.42^{b}	0.45^{a}	0.44^{ab}	0.39^{c}
threonine	0.28^{a}	0.18^{bc}	0.17^{c}	0.21^{b}	0.08^{a}	0.08^{a}	0.07^{a}	0.06^{a}
tryptophan	0.21^{a}	0.12^{a}	0.14^{a}	0.18^{a}	0.40^{b}	0.62^{a}	0.51^{ab}	0.52^{ab}
tyrosine	0.28^{b}	0.45^{ab}	0.72^{a}	0.61^{ab}	0.61^{a}	0.39^{a}	0.42^{a}	0.25^{a}
valine	0.98^{a}	0.63^{b}	0.65^{b}	0.70^{b}	0.29^{a}	0.25^{b}	0.25^{b}	0.22^{c}
**TOAs**:	**23.71^{a}**	**22.63^{a}**	**19.12^{b}**	**13.12^{c}**	**8.40^{a}**	**7.81^{ab}**	**6.95^{b}**	**7.40^{ab}**
citric acid	5.61^{a}	8.91^{b}	8.00^{b}	6.29^{a}	5.33^{a}	4.80^{ab}	4.20^{b}	4.76^{ab}
fumaric acid	0.08^{a}	0.06^{ab}	0.06^{b}	0.06^{b}	0.40^{a}	0.43^{a}	0.38^{a}	0.37^{a}
lactic acid	1.83^{a}	1.23^{b}	0.89^{c}	0.62^{d}	0.13^{a}	0.13^{a}	0.11^{a}	0.13^{a}
malic acid	16.05^{a}	12.25^{b}	10.01^{c}	6.00^{d}	2.38^{a}	2.30^{a}	2.11^{b}	1.99^{b}
oxalic acid	0.12^{a}	0.11^{a}	0.11^{a}	0.11^{a}	0.06^{ab}	0.05^{ab}	0.05^{b}	0.06^{a}
propionic acid	0.03^{b}	0.06^{a}	0.05^{a}	0.04^{ab}	0.11^{a}	0.10^{a}	0.09^{a}	0.09^{a}
**TRCs:**	**6.87^{a}**	**5.82^{b}**	**6.05^{b}**	**5.61^{b}**	**5.49^{a}**	**5.73^{a}**	**5.71^{a}**	**5.45^{a}**
phosphoric acid	6.60^{a}	5.62^{b}	5.90^{b}	5.51^{b}	5.47^{a}	5.72^{a}	5.70^{a}	5.44^{a}
urea	0.26^{a}	0.20^{b}	0.15^{c}	0.11^{d}	0.01^{a}	0.01^{a}	0.01^{a}	0.01^{a}

*- double distilled water - control

Table S9. The concentration of total identified polar metabolites (**TIPMs**), including total soluble carbohydrates (**TSCs**), total amino acids (**TAAs**), total organic acids (**TOAs**), and total remaining compounds (**TRCs**) in roots and coleoptile of 3-day-old seedlings of wheat (*Triticum aestivum* L.) cv. Ostka Strzelecka developed in water suspensions of ZnO NPs at a concentration of 0, 100, 250 and 1000 mg/L. Values (in mg/g DW) are means of 3 replicates. The same letters by the values indicate no statistically significant ($p < 0.05$) differences (valid for roots and coleoptiles separately) based on ANOVA analysis and Tukey's post- hoc corrections.

Metabolites	**Roots**				**Coleoptile**			
	ZnO NPs, mg/L							
	0*	100	250	1000	0*	100	250	1000
**TIPMs:**	**117.57^{ab}**	**114.81^{b}**	**122.20^{a}**	**124.10^{a}**	**133.41^{a}**	**127.49^{a}**	**123.26^{ab}**	**106.46^{b}**
**TSCs**:	**64.88^{c}**	**73.99^{b}**	**82.47^{a}**	**83.98^{a}**	**100.99^{a}**	**98.39^{a}**	**96.85^{a}**	**71.69^{b}**
fructose	10.68^{b}	13.71^{a}	11.27^{ab}	9.90^{b}	28.12^{a}	30.09^{a}	31.73^{a}	18.22^{b}
galactose	4.25^{a}	3.62^{ab}	3.16^{bc}	2.65^{c}	9.18^{a}	8.30^{a}	9.88^{a}	11.89^{a}
glucose	21.57^{a}	16.91^{b}	14.62^{bc}	12.16^{c}	41.83^{a}	39.80^{a}	40.52^{a}	21.93^{b}
1-kestose	4.63^{c}	7.05^{b}	8.42^{a}	7.27^{b}	7.49^{a}	7.96^{a}	4.82^{b}	7.71^{a}
maltose	0.16^{a}	0.16^{a}	0.14^{a}	0.12^{a}	0.06^{a}	0.05^{a}	0.04^{a}	0.07^{a}
myo-inositol	0.28^{a}	0.28^{a}	0.27^{a}	0.28^{a}	1.32^{a}	1.33^{a}	1.36^{a}	1.32^{a}
sucrose	23.31^{d}	32.26^{c}	44.58^{b}	51.61^{a}	12.97^{a}	10.87^{b}	8.51^{c}	10.56^{b}
**TAAs**:	**24.56^{a}**	**18.49^{b}**	**18.50^{b}**	**18.47^{b}**	**15.91^{a}**	**12.62^{ab}**	**8.68^{b}**	**12.65^{ab}**
alanine	1.08^{b}	0.73^{c}	0.98^{b}	1.79^{a}	0.62^{ab}	0.49^{b}	0.29^{c}	0.80^{a}
asparagine	5.83^{a}	4.09^{b}	3.89^{b}	3.00^{c}	2.70^{a}	1.70^{b}	0.68^{c}	1.29^{bc}
aspartic acid	0.21^{b}	0.45^{a}	0.43^{a}	0.20^{b}	0.72^{a}	0.70^{a}	0.42^{a}	0.33^{a}
GABA	0.46^{b}	0.51^{ab}	0.55^{a}	0.52^{ab}	1.71^{a}	1.89^{a}	1.81^{a}	2.33^{a}
glutamic acid	1.48^{a}	1.61^{a}	1.51^{a}	1.81^{a}	1.12^{a}	0.65^{b}	0.15^{c}	0.83^{ab}
glutamine	3.87^{a}	1.94^{b}	1.62^{b}	1.01^{c}	0.70^{a}	0.49^{ab}	0.29^{b}	0.48^{ab}
glycine	0.61^{a}	0.51^{c}	0.57^{ab}	0.54^{bc}	0.84^{a}	0.72^{ab}	0.63^{b}	0.84^{a}
hydroxyproline	2.88^{a}	2.52^{a}	2.55^{a}	2.38^{a}	2.55^{a}	1.79^{a}	0.82^{b}	1.80^{a}
isoleucine	1.29^{a}	0.70^{b}	0.70^{b}	0.77^{b}	0.29^{a}	0.25^{ab}	0.21^{b}	0.20^{b}
leucine	0.52^{a}	0.48^{a}	0.46^{a}	0.46^{a}	0.14^{b}	0.14^{b}	0.21^{a}	0.15^{b}
lysine	0.96^{a}	0.70^{a}	0.83^{a}	0.87^{a}	1.51^{a}	1.07^{a}	0.89^{a}	0.94^{a}
methionine	0.08^{b}	0.02^{c}	0.02^{bc}	0.24^{a}	0.03^{b}	0.04^{ab}	0.03^{b}	0.05^{a}
phenylalanine	0.29^{a}	0.30^{a}	0.34^{a}	0.31^{a}	0.16^{a}	0.13^{ab}	0.09^{b}	0.13^{ab}
proline	1.45^{b}	1.45^{b}	1.55^{b}	1.95^{a}	0.60^{a}	0.66^{a}	0.52^{a}	0.74^{a}
serine	1.07^{a}	0.82^{b}	0.88^{b}	1.03^{a}	0.89^{a}	0.74^{ab}	0.58^{b}	0.75^{a}
threonine	0.53^{a}	0.31^{b}	0.29^{b}	0.33^{b}	0.17^{a}	0.12^{a}	0.04^{b}	0.14^{a}
tryptophan	0.00	0.00	0.00	0.00	0.00	0.00	0.00	0.00
tyrosine	0.50^{a}	0.48^{a}	0.46^{ab}	0.26^{b}	0.67^{a}	0.66^{a}	0.69^{a}	0.48^{a}
valine	1.46^{a}	0.87^{c}	0.87^{c}	1.00^{b}	0.48^{a}	0.38^{b}	0.34^{b}	0.37^{b}
**TOAs**:	**20.38^{a}**	**16.26^{b}**	**14.62^{c}**	**14.85^{c}**	**10.13^{b}**	**9.91^{b}**	**10.41^{b}**	**13.61^{a}**
citric acid	4.90^{b}	5.03^{b}	5.02^{b}	5.67^{a}	6.22^{b}	5.69^{b}	6.34^{b}	8.13^{a}
fumaric acid	0.07^{a}	0.07^{ab}	0.06^{b}	0.06^{ab}	0.49^{b}	0.59^{ab}	0.47^{b}	0.66^{a}
lactic acid	2.70^{a}	1.15^{bc}	1.32^{b}	0.96^{c}	0.17^{bc}	0.13^{c}	0.29^{a}	0.21^{b}
malic acid	12.48^{a}	9.81^{b}	8.03^{c}	7.94^{c}	2.92^{b}	3.20^{b}	3.09^{b}	4.25^{a}
oxalic acid	0.20^{a}	0.16^{a}	0.16^{a}	0.19^{a}	0.20^{a}	0.18^{a}	0.11^{a}	0.23^{a}
propionic acid	0.04^{ab}	0.04^{a}	0.04^{ab}	0.03^{b}	0.14^{a}	0.13^{a}	0.10^{a}	0.13^{a}
**TRCs:**	**7.74^{a}**	**6.06^{c}**	**6.60^{b}**	**6.81^{b}**	**6.37^{c}**	**6.56^{bc}**	**7.32^{b}**	**8.51^{a}**
phosphoric acid	7.46^{a}	5.89^{c}	6.44^{b}	6.67^{b}	6.36^{c}	6.56^{bc}	7.30^{b}	8.49^{a}
urea	0.28^{a}	0.17^{b}	0.17^{b}	0.13^{b}	0.01^{b}	0.01^{b}	0.01^{a}	0.01^{a}

*- double distilled water - control

Table S10. The concentration of total identified polar metabolites (**TIPMs**), including total soluble carbohydrates (**TSCs**), total amino acids (**TAAs**), total organic acids (**TOAs**), and total remaining compounds (**TRCs**) in endosperm of 3-day-old seedlings of wheat (*Triticum aestivum* L.) cv. Collada and cv. Ostka Strzelecka developed in water suspensions of ZnO NPs at a concentration of 0, 100, 250 and 1000 mg/L. Values (in mg/g DW) are means of 3 replicates. The same letters by the values indicate no statistically significant ($p < 0.05$) differences (valid for cultivars separately) based on ANOVA analysis and Tukey's post- hoc corrections.

**Metabolites**	**cv. Collada**				**cv. Ostka Strzelecka**			
	ZnO NPs, mg/L							
	0*	100	250	1000	0*	100	250	1000
**TIPMs**:	**60.51[a]**	**53.56[bc]**	**55.09[b]**	**52.13[c]**	**50.33[a]**	**46.81[ab]**	**41.53[c]**	**44.61[bc]**
**TSCs**:	**56.13[a]**	**49.46[bc]**	**51.17[b]**	**48.04[c]**	**45.81[a]**	**42.82[ab]**	**38.08[c]**	**40.99[bc]**
fructose	0.39[b]	0.43[a]	0.33[c]	0.38[b]	0.36[a]	0.29[b]	0.29[b]	0.25[c]
galactose	0.08[a]	0.06[b]	0.07[ab]	0.07[ab]	0.04[a]	0.04[a]	0.04[a]	0.02[a]
glucose	9.34[ab]	8.92[ab]	9.40[a]	8.77[b]	6.51[a]	6.10[b]	6.45[ab]	5.65[c]
1-kestose	2.52[c]	2.75[ab]	2.63[bc]	2.85[a]	2.44[b]	2.51[b]	2.56[ab]	2.74[a]
maltose	34.31[a]	26.75[b]	28.62[b]	25.81[b]	26.17[a]	22.85[b]	17.05[c]	20.81[b]
maltotriose	1.72[a]	1.29[bc]	1.45[b]	1.26[c]	1.51[a]	1.21[ab]	0.76[b]	0.99[b]
myo-inositol	0.23[a]	0.23[a]	0.24[a]	0.24[a]	0.24[a]	0.22[ab]	0.21[b]	0.21[b]
sucrose	7.54[b]	9.03[a]	8.43[a]	8.65[a]	8.55[b]	9.60[ab]	10.72[a]	10.31[a]
**TAAs**:	**2.77[a]**	**2.66[ab]**	**2.53[b]**	**2.61[ab]**	**2.85[a]**	**2.43[b]**	**2.05[c]**	**2.18[c]**
alanine	0.09[a]	0.09[a]	0.07[a]	0.08[a]	0.13[a]	0.10[ab]	0.08[b]	0.12[ab]
asparagine	0.06[a]	0.05[a]	0.03[b]	0.02[b]	0.03[b]	0.02[c]	0.04[a]	0.03[bc]
aspartic acid	0.01[a]	0.01[a]	0.01[a]	0.01[a]	0.01[a]	0.01[a]	0.01[a]	0.01[a]
GABA	0.08[c]	0.09[b]	0.09[b]	0.11[a]	0.11[a]	0.11[a]	0.08[b]	0.10[ab]
glutamic acid	0.22[a]	0.22[a]	0.20[b]	0.21[ab]	0.20[a]	0.16[ab]	0.09[c]	0.16[b]
glutamine	0.09[a]	0.09[a]	0.07[a]	0.08[a]	0.05[a]	0.06[a]	0.09[a]	0.05[a]
glycine	0.10[a]	0.09[b]	0.09[b]	0.09[b]	0.13[a]	0.10[b]	0.09[b]	0.10[b]
hydroxyproline	0.37[a]	0.38[a]	0.31[b]	0.37[a]	0.41[a]	0.34[ab]	0.22[c]	0.30[bc]
isoleucine	0.10[a]	0.09[bc]	0.09[c]	0.09[ac]	0.13[a]	0.10[b]	0.10[b]	0.10[b]
leucine	0.17[a]	0.15[b]	0.15[b]	0.14[b]	0.16[ab]	0.15[ab]	0.18[a]	0.13[b]
lysine	0.22[a]	0.19[a]	0.23[a]	0.21[a]	0.15[a]	0.15[a]	0.10[b]	0.10[b]
methionine	0.01[a]	0.01[a]	0.01[a]	0.01[a]	0.01[a]	0.01[a]	0.01[a]	0.01[a]
phenylalanine	0.28[a]	0.25[b]	0.23[b]	0.25[b]	0.30[a]	0.23[b]	0.20[b]	0.21[b]
proline	0.38[a]	0.38[a]	0.34[b]	0.35[ab]	0.45[a]	0.39[b]	0.38[b]	0.36[b]
serine	0.13[a]	0.13[a]	0.12[a]	0.12[a]	0.16[a]	0.13[b]	0.13[b]	0.13[b]
threonine	0.02[a]	0.03[a]	0.02[b]	0.02[ab]	0.04[a]	0.02[b]	0.01[b]	0.02[b]
tryptophan	0.12[b]	0.14[ab]	0.17[a]	0.15[ab]	0.09[a]	0.06[b]	0.02[c]	0.05[b]
tyrosine	0.15[a]	0.13[a]	0.16[a]	0.14[a]	0.09[ab]	0.10[a]	0.04[b]	0.06[ab]
valine	0.18[a]	0.15[b]	0.15[b]	0.16[b]	0.22[a]	0.17[b]	0.18[b]	0.16[b]
**TOAs**:	**0.95[a]**	**0.83[b]**	**0.84[b]**	**0.86[b]**	**0.91[a]**	**0.95[a]**	**0.89[a]**	**0.88[a]**
citric acid	0.54[a]	0.44[b]	0.46[b]	0.46[b]	0.55[a]	0.58[a]	0.56[a]	0.54[a]
fumaric acid	0.01[a]	0.01[a]	0.01[a]	0.01[a]	0.007[a]	0.006[a]	0.003[c]	0.005[b]
lactic acid	0.17[a]	0.12[b]	0.12[b]	0.13[ab]	0.10[a]	0.08[a]	0.10[a]	0.08[a]
malic acid	0.12[b]	0.15[a]	0.14[a]	0.14[a]	0.12[a]	0.12[a]	0.09[b]	0.10[b]
oxalic acid	0.10[b]	0.10[ab]	0.10[ab]	0.12[a]	0.13[b]	0.16[a]	0.14[b]	0.15[ab]
propionic acid	0.003[b]	0.004[ab]	0.004[a]	0.004[a]	0.01[a]	0.00[a]	0.01[a]	0.01[a]
**TRCs**:	**0.66[a]**	**0.61[ab]**	**0.56[b]**	**0.62[a]**	**0.76[a]**	**0.62[b]**	**0.51[bc]**	**0.55[c]**
phosphoric acid	0.65[a]	0.60[ab]	0.55[b]	0.61[a]	0.75[a]	0.61[b]	0.51[bc]	0.54[c]
urea	0.01[a]	0.01[a]	0.01[a]	0.01[a]	0.01[a]	0.01[a]	0.01[a]	0.01[a]

*- double distilled water - control

Table S11. Pearson correlation coefficients for Pearson's correlation of sucrose with glucose, fructose, galactose and monosaccharides (sum of glucose, fructose and galactose) in epicotyl and root of pea (*Pisum sativum* L.) seedlings cv. Nemo and Tarchalska.

cultivar	seedling part	sucrose × glucose	sucrose × fructose	sucrose × galactose	sucrose × monosaccharides
**Nemo**	root	-0.07	-0,4	-0.49	-0.26
	epicotyl	-0.44	0,52	-0.36	-0.41
**Tarchalska**	root	-0.88*	-0,86*	-0.88*	-0.88*
	epicotyl	-0.90*	-0,80*	-0.87*	-0.93*

* - p > 0.01

Table S12. Pearson correlation coefficients for Pearson's correlation of sum of sucrose plus 1-kestose with sum of glucose plus fructose in coleoptile and roots of wheat (*Triticum aestivum* L.) seedlings cv. Collada and Ostka Strzelecka.

cultivar	seedling part	sucrose + 1-kestose × glucose + fructose
**Collada**	root	0.72*
	coleoptiles	0.01
**Ostka Strzelecka**	root	-0.90*
	coleoptiles	-0.10

* - p > 0.01

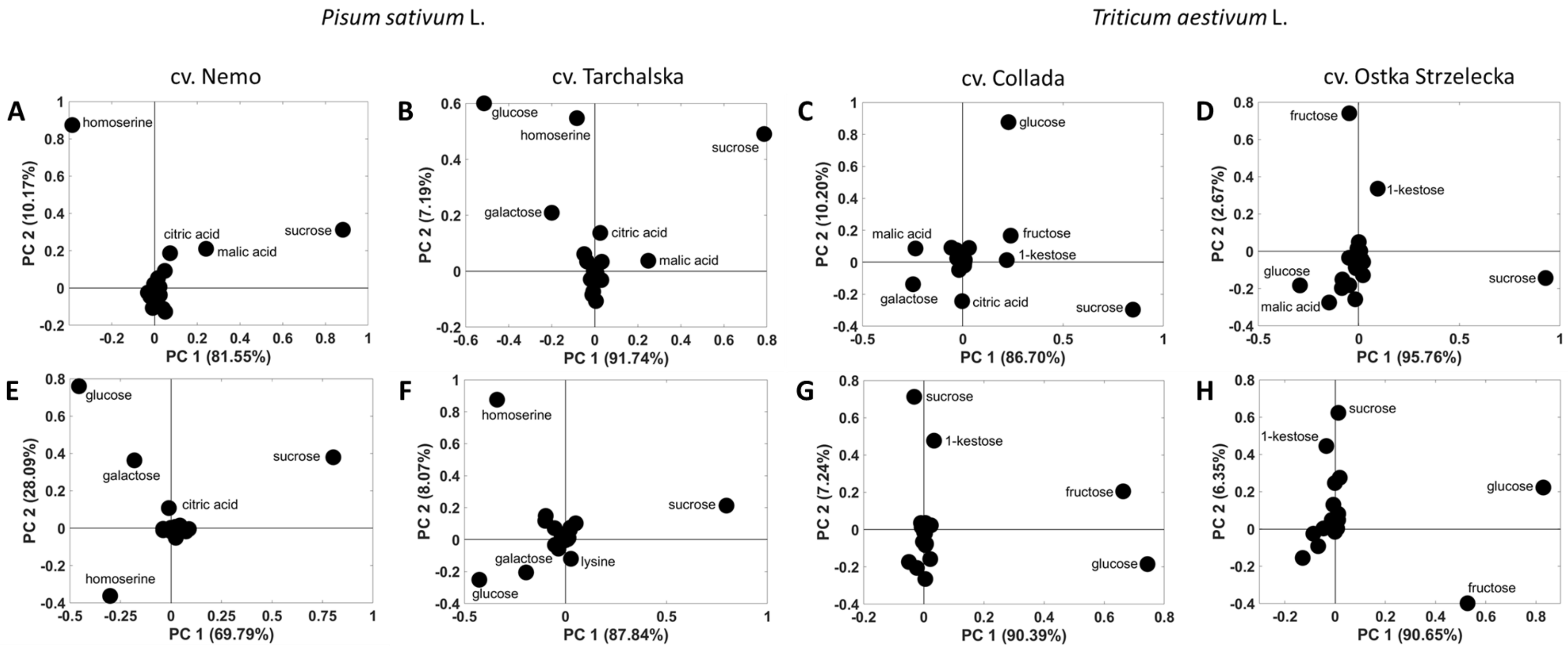

Figure S2. PCA loadings plots of polar metabolites of roots (A, B) and epicotyls (E, F) of 4-day-old seedlings of pea (*Pisum sativum* L.) and roots (C, D) and coleoptile (G, H) of 3-day-old seedlings of wheat (*Triticum aestivum* L.) developed in suspension of ZnO NPs at 0, 100, 250 and 1000 mg/L.

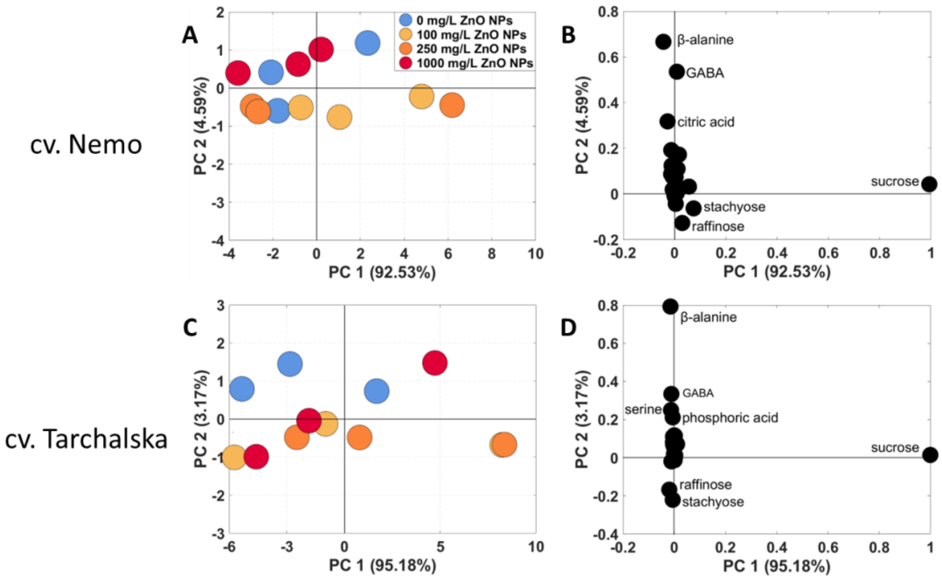

Figure S3. PCA of metabolic profiles of cotyledons of 4-day-old seedlings of pea (*Pisum sativum* L.) cv. Nemo (A) and Tarchalska (C), developed in suspension of ZnO NPs at 0, 100, 250 and 1000 mg/L and PCA loadings plots of polar metabolites (B, D for cv. Nemo and cv. Tarchalska, respectively).

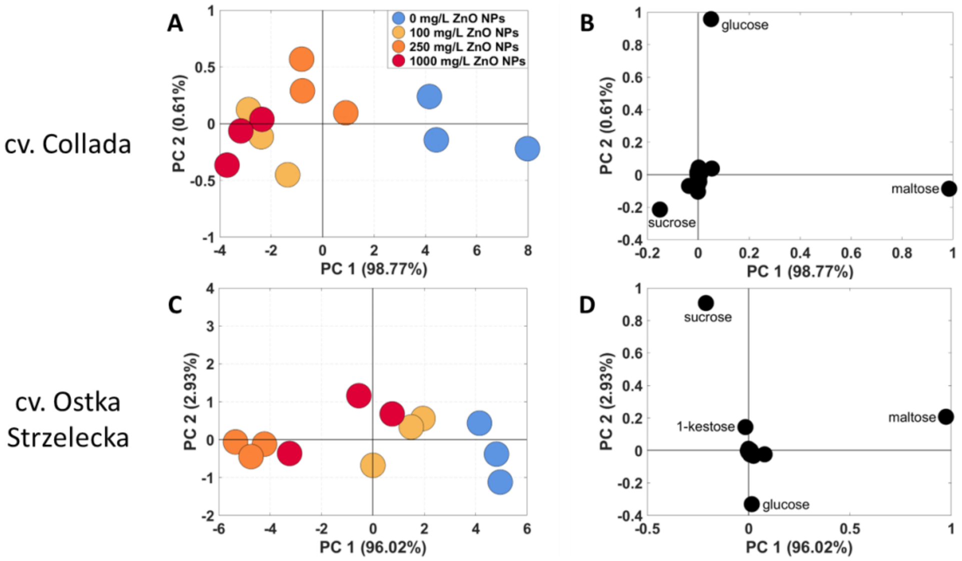

Figure S4. PCA of metabolic profiles of endosperm of 3-day-old seedlings of wheat (*Triticum aestivum* L.) cv. Collada (A) and cv. Ostka Strzelcka (C), developed in ZnO NPs suspension at 0, 100, 250 and 1000 mg/L and PCA loadings plots of polar metabolites (B, D for cv. Collada and cv. Ostka Strzelecka, respectively).
